# On the evolution of the quality of macromolecular models in the PDB

**DOI:** 10.1111/febs.15314

**Published:** 2020-04-20

**Authors:** Dariusz Brzezinski, Zbigniew Dauter, Wladek Minor, Mariusz Jaskolski

**Affiliations:** ^1^ Center for Biocrystallographic Research Institute of Bioorganic Chemistry Polish Academy of Sciences Poznan Poland; ^2^ Institute of Computing Science Poznan University of Technology Poland; ^3^ Center for Artificial Intelligence and Machine Learning Poznan University of Technology Poland; ^4^ Department of Molecular Physiology and Biological Physics University of Virginia Charlottesville VA USA; ^5^ Synchrotron Radiation Research Section Macromolecular Crystallography Laboratory National Cancer Institute Argonne National Laboratory Argonne IL USA; ^6^ Department of Crystallography Faculty of Chemistry A. Mickiewicz University Poznan Poland

**Keywords:** nucleic acids, PDB, proteins, structure quality, X‐ray crystallography

## Abstract

Crystallographic models of biological macromolecules have been ranked using the quality criteria associated with them in the Protein Data Bank (PDB). The outcomes of this quality analysis have been correlated with time and with the journals that published papers based on those models. The results show that the overall quality of PDB structures has substantially improved over the last ten years, but this period of progress was preceded by several years of stagnation or even depression. Moreover, the study shows that the historically observed negative correlation between journal impact and the quality of structural models presented therein seems to disappear as time progresses.

## Graphical abstract

Quality criteria are proposed to rank the macromolecular models in the Protein Data Bank (PDB), and the results are correlated with time and with the journals that published papers based on those models. The overall quality of PDB structures has substantially improved over the last decade, and the negative correlation between journal impact factor and the quality of structural models presented therein seems to disappear as time progresses.

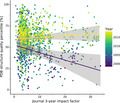

**Abbreviations**
IPP: impact per publication
MAD: median absolute deviation
MAE: mean absolute error
MICE: multiple imputation by chained equations
PCA: principal component analysis
PDB: Protein Data Bank
RMSE: root‐mean‐square error
RSRZ: real‐space *R*‐value *Z*‐score
SNIP: source normalized impact per paper

## Introduction

Structural biology has fulfilled a history‐changing mission in science at the interface of physics, chemistry, and biology when for over six decades it has maintained its leading role in providing the structural basis for our understanding of life [1, 2, 3, 4]. Its results were always regarded as exceptionally solid, and created a gold standard in biological research, almost unattainable in many other areas of life sciences. This view has largely persisted until today, in part even fortified by the incredible technical advances in the generation and detection of X‐rays, progress in computer software development, revolution in biotechnology, and innovations in crystallogenesis. However, with the expansion of the Protein Data Bank (PDB) [5] from merely seven structures at its inception in 1971 to ~ 160 000 today, it is inevitable that some of the macromolecular models will be subpar and sometimes even incorrect. Unfortunately, suboptimal structures have a tangible negative impact on biomedical research that relies on structural data [6]. However, crystallographers, who have always been in the forefront of structural biology, also in this regard seem to be setting example of how to deal with suboptimal or irreproducible science. The protein crystallographic community has been made painfully aware of these problems [7, 8, 9, 10], partly in the rising wave of concern about irreproducibility of scientific research and of biomedical research in particular [11]. This awareness has led to positive outcomes, such as, for example, development of structural validation criteria and protocols, or development of tools for the detection and correction of model errors [12].

The PDB itself, who is the chief custodian of the structural treasury amassed by structural biologists, has been developing tools and standards for the assessment of the quality of the structural models deposited in its archives [13, 14]. Similarly, more and more journals are starting to require structural validation reports generated by the PDB upon manuscript submission. However, in some opinions these actions are still insufficient and many problems could be better checked at source rather than being tracked in time‐delayed model‐correction actions [6, 15], when the ripple effect of structural errors may have already taken its toll. Objectively speaking, however, in view of the immense scale of the PDB, one should be in fact grateful for all the effort already taken and the plans proposed for the future of the data bank. In particular, the PDB has been developing a consistent and informative set of quality indicators, which now accompany each new crystal structure deposition. These indicators have been recently used to assess the evolution of the quality of the PDB deposits with time [16].

However, it is not only the PDB that has the responsibility for maintaining high standard of the structural information generated by structural biology. The prime burden is of course on the authors, but this is usually the weakest link: rarely because of ill intention or fraud and more frequently because of haste, lack of training, lack of supervision, or the delusive belief that the incredible recent progress has converted crystallography to a very easy and almost completely automatic analytical method. An important deal of responsibility rests with the referees and editors of the journals that publish those results, as the ripple effect of error and fatal contamination of science are most efficiently propagated through cited literature [17]. More than a decade ago, Brown and Ramaswamy (hereinafter B&R) published a survey of the quality of crystallographic models of biological macromolecules [18] and correlated the results with the journals in which those models had been published. The results came as a bit of a shock to many because it turned out that the journals usually regarded as the most prestigious were found to publish worse than average structures when compared to other journals. The *FEBS Journal* was one of the first to request structure validation reports and thus in the ranking list of B&R was among the top journals. Similar questions have been raised by Read and Kleywegt (hereinafter R&K), albeit using different statistical tools [19]. In contrast to the B&R study, R&K reported very small quality differences between structures published in high‐impact journals and in other venues.

Nearly 13 years after the B&R study and with the PDB expanded nearly four times, we decided to conduct a similar analysis to see whether the community at large, or at least its journals, have improved. In our approach, we used the statistical methods of data imputation and principal component analysis (PCA) of the model quality indicators recommended by the PDB. In contrast to previous studies, which focused on protein structures only, our analysis comprises all crystallographic structures in the PDB, that is, also includes nucleic acids. Moreover, we also consider models marked as *To be published*, which were not analyzed by B&R or R&K. Although the scope of data and the statistical tools we are using are different from those used by B&R in 2007, we are still able to compare the journal rankings of the two surveys because our approach may be easily adapted to a retrospective analysis of data from past versions of the PDB. It is important to clarify that omission of NMR and Cryo‐EM structures was intentional. Considering the difficulties connected with estimation of quality of NMR and Cryo‐EM structural models, and also the very small contribution of both these methods to the characterization of structures that contain ligands (and are thus most interesting and important), we decided to focus on models provided by X‐ray crystallography, which represent 89% of all models currently deposited in the PDB.

Our results show that the overall quality of PDB structures has substantially improved over the last 10 years. However, our study also shows that this period of improvement was preceded by several years of stagnation or, if one considers the improvement of software and hardware over time, even depression. Finally, the observation made by B&R that journal impact factor (reputation) is frequently negatively correlated with structure quality is no longer true.

## Results

### Measure of overall model quality and missing data imputation

The analysis included all X‐ray structures available in the PDB as of December 10, 2019, totaling 141 154 deposits dating back as far as 1972. To assess the quality of structures published in particular journals, we initially attempted to use the *Q*1_p_ measure proposed by Shao *et al.* [16]. *Q*1_p_ is a measure of overall protein structure quality that combines into one number five different indicators: *R*_free_, RSRZ (normalized real‐space *R*‐factor) outliers, Ramachandran outliers, Rotamer outliers, and Clashscore [20] using the following formula:

(1) $$Q1_{p}=\frac{P_{R_{\text{free}}}+P_{\%\text{RSRZ}}+P_{\text{PC}1(\text{geometry})}}{3},$$

where $P_{R_{\text{free}}}$ , *P*_%RSRZ_, and *P*_PC1(geometry)_ are ranking percentiles (the higher the better), characterizing for a given structural model, respectively, its *R*_free_, percentage of RSRZ outliers, and the first principal component of the PCA of Ramachandran outliers, Rotamer outliers, and Clashscore (see Methods section for details). Once *Q*1_p_ is calculated, each PDB deposit is ranked within the population to obtain its final ranking percentile $P_{Q1_{p}}$ , with the lowest (worst) value of *Q*1_p_ at 0% and highest (best) at 100% [16]. We note that in this paper we took an averaging approach to percentiles, that is, a group of tied *Q*1_p_ values was assigned the same percentile rank, one that is the average rank of the group. By combining five distinct quality measures, $P_{Q1_{p}}$ provides a simple way of comprehensive comparison and ranking of many structural models.

The $P_{Q1_{p}}$ metric was originally designed to assess protein structures only. For nucleic acid structures, which are also present in the PDB, *Q*1_p_ cannot be used directly because the notions of Ramachandran and Rotamer outliers are not applicable to those structures. However, for proteins both missing elements are implicitly contained in *P*_PC1(geometry)_. Therefore, for nucleic acids we calculated analogous *Q*1_n_ without the use of PCA, but applying the following simplified formula:

(2) $$Q1_{n}=\frac{P_{R_{\text{free}}}+P_{\%\text{RSRZ}}+P_{\text{Clashscore}}}{3},$$

where *P*_Clashscore_ is the ranking percentile of Clashscore. In the following analysis, *Q*1_p_ and *Q*1_n_ (and, consequently, $P_{Q1_{p}}$ and $P_{Q1_{n}}$ ) were computed separately for proteins and nucleic acids, respectively. This way, the percentiles were used for *Q*1_p_ and *Q*1_n_ rank structures of the respective type. Protein–nucleic acid complexes were assigned to the protein group, since it is possible to calculate all quality metrics for such structures.

Since averaging of multiple quality metrics might potentially blur the spotlight on models with serious problems, an alternative aggregation method could involve taking only the minimum percentile of all the metrics used. In this approach, a structure is considered as good as its weakest feature, according to the following formulas:

(3) $$Q1_{{\text{p}}_{{}_{\min}}}=\min\left(P_{R_{\text{free}}},P_{\%\text{RSRZ}},P_{\text{PC}1(\text{geometry})}\right),$$

(4) $$Q1_{n_{\min}}=\min\left(P_{R_{\text{free}}},P_{\%\text{RSRZ}},P_{\text{Clashscore}}\right).$$

In the remainder of the paper, we will focus mainly on the averaging approach using Eqns (1, 2), but will also compare it with the *minimum approach* based on Eqns (3,  4).

It must be emphasized that $P_{Q1_{p}}$ can be computed only for those PDB structures that have *all five* (or in the case of $P_{Q1_{n}}$ *all three*) component measures attached to them. The PDB has done an excellent job of calculating these metrics for most of the deposits, but not all structures have all the necessary data to perform these calculations. Overall, 12.7% of all considered deposits are missing at least one quality metric, with RSRZ being the dominating missing value (Table 1). Leaving this situation as is would effectively limit the analysis to structures published after 1992, that is, to the time after *R*_free_ was introduced [21]. To circumvent this dilemma and to perform a study encompassing the entire timespan of the PDB, we have developed a protocol for the estimation of the missing values based on a machine‐learning data imputation method.

**Table 1:** Quality metric means, standard deviations, and fractions of missing values in the PDB.

	Mean	Standard deviation	Missing values (%)
Metric			
Clashscore	8.05	9.11	0.10
Ramachandran outliers (%)	0.49	1.26	1.69
Rotamer outliers (%)	3.26	3.65	1.72
RSRZ outliers (%)	4.05	4.06	9.56
*R*_free_ (%)	23.35	3.82	4.29
Supporting metrics			
*R* (%)	19.31	3.25	2.39
Resolution (Å)	2.13	0.56	0
Year of deposition	–	–	0

The validity of the data imputation procedure was assessed on the complete portion of the PDB, to which artificially missing (i.e., deliberately removed) values were introduced at random following the missing data proportions of each metric. The missing values were then replaced using either the metric's mean, median, or by an iterative method called multiple imputation by chained equations (MICE) [22, 23] with Bayesian linear regression [24]. MICE builds regression functions for subsequent metrics based on nonmissing values from other variables. The variables used to aid imputation involved all the metrics in question, plus three supporting variables, not used in the assessment protocol: the *R*‐factor, data resolution (*d*_min_), and year of deposition (Table 1). The results of 100 random experiments testing the imputation methods are presented in Table 2.

**Table 2:** Evaluation of data imputation methods. Mean results of 100 random experiments with standard deviations given in parentheses, in units of the last significant digit of the mean.

Error	Method	Clashscore	RSRZ outliers (%)	Ramachandran outliers (%)	Rotamer outliers (%)	*R*_free_ (%)
MAD	MICE	**2.5 (3)**	2.01 (2)	0.21 (1)	**1.23 (3)**	**1.02 (2)**
	Mean	4.4 (3)	2.30 (2)	0.49 (1)	2.11 (4)	2.50 (4)
	Median	2.9 (3)	**1.86 (2)**	**0.04 (1)**	1.39 (4)	2.50 (4)
MAE	MICE	**3.7 (4)**	**2.54 (3)**	**0.41 (2)**	**1.77 (4)**	**1.31 (2)**
	Mean	5.7 (6)	2.74 (3)	0.61 (2)	2.56 (6)	3.00 (3)
	Median	5.1 (7)	2.60 (3)	0.49 (3)	2.34 (7)	3.00 (3)
RMSE	MICE	**5.7 (15)**	**3.84 (15)**	**0.84 (11)**	**2.64 (10)**	**1.77 (3)**
	Mean	8.9 (23)	4.04 (14)	1.24 (16)	3.65 (14)	3.82 (4)
	Median	9.3(24)	4.17 (14)	1.32 (16)	3.85 (15)	3.82 (4)

Best values for each error estimation method are given in bold. MAD, median absolute deviation; MAE, mean absolute error; RMSE, root‐mean‐square error.

It can be seen that MICE is superior to mean/median replacement for all metrics according to the mean absolute error (MAE) and root‐mean‐square error (RMSE), and for all but two metrics according to the median absolute deviation (MAD). All the differences between MICE and the remaining methods are statistically significant according to the Friedman and Nemenyi *post hoc* tests [25] (*p* < 0.001). In terms of absolute values, the MAE of MICE is usually two to four times smaller than the standard deviation of a given quality metric (Table 1, Fig. S1). The results are particularly good for Clashscore and *R*_free_, owing to the small number of missing values and high correlation with *R*, respectively. In the remaining part of the paper, we discuss results obtained for the full PDB dataset with missing values imputed using the MICE method. We want to stress that in doing so our goal is to give an approximate overview of the average quality of structures in the early years of the PDB, and not to provide a way to assess individual deposits with missing quality metrics or to create nonexistent data.

### Model quality at the time of deposition

**Fig. 1:**
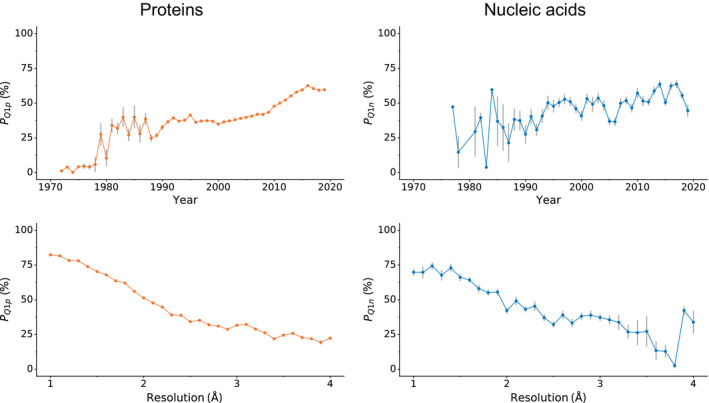
*P_Q_*_1_ analysis. Variation in the mean *P_Q_*_1_ percentile (higher is better) over time (top) and as a function of resolution (bottom) for proteins (left) and nucleic acids (right). Error bars indicate estimated unbiased standard errors of the mean.

Figure 1 shows that $P_{Q1_{p}}$ and $P_{Q1_{n}}$ tend to gradually improve over the years. Almost identical trends can be noticed when looking at deposits without imputed data (Fig. S2) and when using the minimum approach (Fig. S3). Obviously, this trend is correlated with the advances in the generation of X‐rays and in data collection procedures, with better computer hardware and software, with heightened structural validation standards, and with progress in crystallogenesis. If one were to use *P_Q_*_1_ (i.e., $P_{Q1_{p}}$ or $P_{Q1_{n}}$ depending on structure type) calculated over all the analyzed years to rank journals, then journals with longer history would be at a disadvantage because they contain old, quality‐wise inferior structures. Thus, even though a structure might have been refined to an impressively high standard in its time, today it might be treated as a poorly refined case. One could, of course, recalculate the percentiles separately for each decade or even shorter time periods, but this might not be enough to cure this problem (see the rapid improvement in quality over the last 10 years) or could drastically reduce the data volume and effectively make journal comparisons impossible. Therefore, we introduce here a new, time (*t*)‐dependent *P_Q_*_1_(*t*) parameter, which corresponds to *P_Q_*_1_ calculated at the time of structure deposition. For example, the 1990 PDB deposition 2RSP [26] achieves an overall quality percentile *P_Q_*_1_ of 36%, meaning that it is better than only 36% protein deposits that are currently held in the archive. Should the structure be ranked against the 416 structures deposited prior to 2RSP, it achieves *P_Q_*_1_(*t*) of 69%, meaning that it was significantly above‐average at the time of its deposition.

Moreover, in view of the very high correlation between quality and resolution (Fig. 1, Figs S2 and S3), we propose yet another measure, called *P_Q_*_1_(*t*,*d*)*. P_Q_*_1_(*t*,*d*)* is* the *Q1* percentile calculated at the time of structure deposition (*t*) for a given resolution interval (*d*), where the resolution is rounded to the nearest 0.1 Å and capped at 1 and 4 Å. The 2RSP structure from the previous example scores a *P_Q_*_1_(*t*,*d*) of 75%. The advantage of using *P_Q_*_1_(*t*,*d*) is that data resolution will not affect the journal ranking list.

**Fig. 2:**
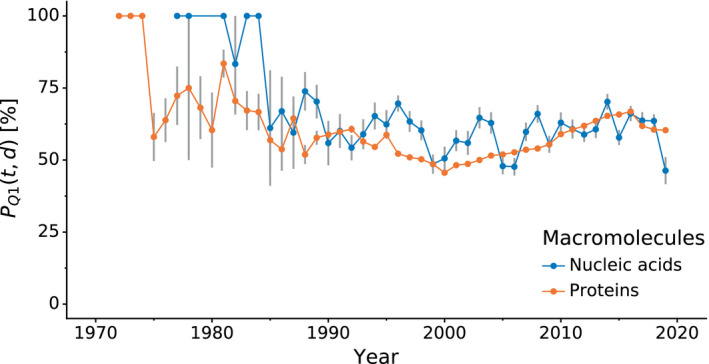
Comparison of *P_Q_*_1_(*t*,*d*) of protein and nucleic acid structures over time. Variation in mean *P_Q_*_1_(*t*,*d*) quality percentile (*y*‐axis, higher is better), comparing nucleic acid and protein structures (color) over time (*x*‐axis). Error bars indicate estimated unbiased standard errors of the mean.

Using *P_Q_*_1p_(*t*,*d*) and *P_Q_*_1n_(*t*,*d*), one can assess the quality of protein and nucleic acid models over time. The average *P_Q1_*_p_(*t*,*d*) for proteins in the PDB is 58.7%, whereas nucleic acids have the average *P_Q1_*_n_(*t*,*d*) of 59.9%. Figure 2 shows how the model quality at the time of deposition of these two types of macromolecules has evolved over the years. For many years in the past, newly deposited nucleic acid models were usually of better quality than newly deposited protein models, especially between 1993 and 2004. However, the steady improvement of the quality of protein models in the last decade has made them currently to be on a par, if not better, than currently deposited nucleic acid models. Similar trends were observed using $P_{Q1_{p_{\min}}}\left(t,d\right)$ and $P_{Q1_{n_{\min}}}\left(t,d\right)$ , that is, the minimum approach (Fig. S4).

In the following subsections, we will focus on ranking structures and their corresponding journals according to *P_Q_*_1_(*t*,*d*). The rankings associated with *P_Q_*_1_(*t*), $P_{Q1_{\min}}\left(t,d\right)$ , and $P_{Q1_{\min}}\left(t\right)$ are available in the online supplementary materials for this publication. For the purposes of ranking journals, the percentiles for proteins and nucleic acids will be combined and denoted jointly as *P_Q_*_1_(*t*,*d*) or *P_Q_*_1_(*t*).

### All‐time journal ranking

Out of 800 unique journals being the primary citations for the 141 154 deposits found in the PDB, we selected those that published papers presenting at least 100 macromolecular structures. We decided to limit the list of journals to such a subset, as we believe that it may be too early to assess journals with less than 100 described structures. The resulting 91 journals were ranked according to average *P_Q_*_1_(*t*,*d*) (Table 3) as well as *P_Q_*_1_(*t*), $P_{Q1_{\min}}\left(t,d\right)$ , and $P_{Q1_{\min}}\left(t\right)$ (Tables S1–S3).

**Table 3:** All‐time journal ranking according to *P_Q_*_1_(*t*,*d*). The ranking includes all the journals that had at least 100 primary citations of structures in the PDB. *P_Q_*_1_(*t*,*d*) higher than 50% means that the structures published in a given journal were, on average, better than 50% of structures of similar resolution present in the PDB at the time of deposition. Journals with more than 1000 structures are highlighted in gray. The most frequent venue (*To be published*) is highlighted in bold.

Rank	Journal	Mean *P_Q_*_1_(*t*,*d*) (%)	Mean resolution (Å)	*G*‐mean resolution (Å)	*V*‐mean resolution (Å)	Structure count
1	*Tuberculosis (Edinb)* ^a^	87.38	2.02	2.00	1.92	132
2	*Eur J Med Chem* ^a^	71.05	2.03	1.97	1.81	418
3	*ACS Catal* ^a^	69.25	1.94	1.88	1.70	241
4	*ACS Infect Dis* ^a^	68.32	1.95	1.90	1.78	153
5	*Chembiochem* ^a^	68.30	1.92	1.87	1.71	527
6	*IUCrJ* ^a^	67.76	1.95	1.88	1.69	281
7	*Org Biomol Chem*	67.42	1.88	1.82	1.64	167
8	*mBio*	66.72	2.24	2.15	1.91	169
9	*Arch Biochem Biophys* ^a^	66.13	2.08	2.03	1.89	276
10	*Int J Mol Sci*	65.77	2.12	2.04	1.81	103
11	*Chemistry*	65.66	1.82	1.77	1.62	242
12	*Biochem J* ^a^	65.42	2.11	2.06	1.89	1033
13	*FEBS J* ^a^	65.11	2.02	1.97	1.81	1539
14	*Cell Host Microbe*	64.85	2.50	2.43	2.20	107
15	*PLoS Pathog* ^a^	64.72	2.25	2.17	1.96	656
16	*Nat Microbiol*	64.69	2.32	2.25	2.04	111
17	*Chem Commun*	64.58	1.81	1.75	1.59	280
18	*Virology*	64.38	2.44	2.37	2.18	126
19	*Nat Chem Biol* ^a^	64.12	2.19	2.12	1.92	1013
20	*Appl Environ Microbiol*	64.00	2.01	1.96	1.83	112
21	*ACS Chem Biol* ^a^	63.83	2.04	1.98	1.83	1104
22	*Acta Cryst F* ^a^	63.59	2.09	2.02	1.81	1466
23	*Sci Rep* ^a^	63.39	2.16	2.09	1.88	1847
24	*Angew Chem* ^a^	63.25	1.92	1.85	1.64	1065
25	*J Inorg Biochem*	62.82	1.78	1.73	1.57	171
26	*J Biol Inorg Chem*	62.61	1.88	1.82	1.64	265
27	*ACS Omega*	62.60	1.81	1.77	1.64	102
28	*J Comput Aided Mol Des*	62.39	1.88	1.86	1.77	115
29	*Chem Sci*	61.98	1.87	1.83	1.68	268
30	*MAbs*	61.91	2.35	2.29	2.14	115
31	*J Synchrotron Radiat*	61.75	1.79	1.73	1.56	147
32	*PLoS One* ^a^	61.57	2.15	2.09	1.92	2057
33	*Glycobiology*	61.48	1.96	1.90	1.74	188
34	*ChemMedChem*	61.40	1.94	1.88	1.69	556
35	**To Be Published** ^a^	61.39	2.03	1.98	1.81	22 421
36	*Nat Commun* ^a^	61.37	2.21	2.11	1.86	3538
37	*FEBS Lett*	61.30	2.10	2.04	1.85	814
38	*Nat Chem*	61.11	1.95	1.85	1.65	173
39	*Antimicrob Agents Chemother*	61.10	1.96	1.88	1.65	309
40	*ACS Med Chem Lett*	61.01	2.12	2.06	1.88	1062
41	*RNA*	60.95	2.44	2.32	1.97	245
42	*J Am Chem Soc*	60.89	2.00	1.93	1.74	2369
43	*Protein Eng Des Sel*	60.59	2.05	2.00	1.84	294
44	*Cell Chem Biol*	60.17	2.08	2.03	1.88	902
45	*Acta Cryst D*	60.06	1.99	1.91	1.70	4952
46	*Mol Pharmacol*	59.99	2.35	2.27	2.02	129
47	*BMC Struct Biol*	59.56	2.08	2.02	1.84	228
48	*Biochemistry*	59.45	2.05	2.00	1.84	8896
49	*Mol Microbiol*	59.41	2.16	2.09	1.87	412
50	*Biochimie*	59.04	2.07	2.02	1.81	128
51	*J Biochem*	59.01	2.05	2.01	1.88	279
52	*FASEB J*	58.88	2.03	1.98	1.86	161
53	*Nucleic Acids Res*	58.77	2.28	2.21	1.98	2127
54	*Structure*	58.53	2.20	2.11	1.86	5348
55	*Protein Sci*	58.46	2.07	2.02	1.85	2235
56	*J Med Chem*	58.29	2.07	2.02	1.86	5525
57	*J Virol*	58.24	2.34	2.26	2.06	957
58	*Plant Cell*	58.00	2.21	2.17	2.05	138
59	*Biochim Biophys Acta*	57.99	2.09	2.04	1.87	600
60	*Sci Adv*	57.95	2.33	2.22	1.85	182
61	*J Biol Chem*	57.91	2.12	2.06	1.89	11 055
62	*J Struct Biol*	57.49	2.15	2.08	1.90	1038
63	*J Bacteriol*	57.37	2.22	2.16	2.01	371
64	*Bioorg Med Chem*	57.28	2.10	2.04	1.88	659
65	*Proteins*	57.09	2.07	2.01	1.84	1999
66	*Cell Rep*	56.55	2.52	2.42	2.16	399
67	*J Exp Med*	56.35	2.34	2.28	2.08	121
68	*Biochem Biophys Res Commun*	56.28	2.18	2.11	1.92	976
69	PNAS^a^	55.79	2.27	2.19	1.94	7376
70	*Int J Biol Macromol*	55.51	2.05	2.00	1.80	168
71	*PLoS Biol*	55.43	2.35	2.25	2.01	336
72	*eLife*	55.40	2.41	2.30	2.03	869
73	*Biophys J*	55.36	1.92	1.85	1.66	199
74	*J Mol Biol* ^a^	55.25	2.13	2.06	1.88	9507
75	*Cell Res*	54.60	2.42	2.36	2.20	189
76	*J Struct Funct Genom*	54.30	2.07	2.03	1.92	168
77	*Nature* ^a^	53.84	2.52	2.42	2.12	3060
78	*Protein Cell*	53.44	2.26	2.21	2.04	178
79	*Genes Dev*	53.44	2.41	2.33	2.11	279
80	*Science* ^a^	53.15	2.50	2.39	2.10	1949
81	*Nat Immunol*	52.94	2.52	2.45	2.22	119
82	*EMBO Rep*	52.92	2.35	2.28	2.09	211
83	*J Immunol*	52.10	2.26	2.19	2.02	296
84	*Neuron*	51.35	2.63	2.41	2.08	149
85	*Immunity*	51.10	2.44	2.37	2.18	265
86	*Commun Biol*	51.01	2.39	2.31	2.00	104
87	*Mol Cell* ^a^	50.38	2.45	2.37	2.14	1599
88	*Bioorg Med Chem Lett* ^a^	50.19	2.19	2.16	2.05	1590
89	*Nat Struct Mol Biol* ^a^	49.78	2.40	2.31	2.07	2915
90	*Cell* ^a^	49.38	2.54	2.45	2.20	1563
91	*EMBO J* ^a^	49.15	2.37	2.30	2.10	1910

^a^ Journals that have average *P_Q_*_1_(*t*,*d*) significantly different than the average *P_Q_*_1_(*t*,*d*) of the entire PDB, according to Welch's *t*‐test with the Bonferroni correction at significance level α = 0.001. Mean denotes the arithmetic mean, *G*‐mean denotes the geometric mean (log‐average), and *V*‐mean denotes the mean in Å^−3^.

Surprisingly, the first place in all versions of the ranking is occupied by *Tuberculosis*, a venue that is not well known as a structural journal. However, this place is well earned since *Tuberculosis* has over 16 percentage points of advantage over the second ranked journal in terms of *P_Q_*_1_(*t*,*d*) and 12 percentage points of advantage in the *P_Q_*_1_(*t*) ranking. A closer inspection of the structures published in *Tuberculosis* reveals that the vast majority of structures refer to one publication titled ‘Increasing the structural coverage of tuberculosis drug targets’ [27]. The publication and its corresponding structures are the result of the joint effort of various departments working in the Seattle Structural Genomics Center for Infectious Disease. This finding is in accordance with the conclusions of B&R [18] that structural genomics initiatives usually deposit structures of above‐average quality [28, 29]. Indeed, taking into account all 12 494 deposits attributed to structural genomics projects, they achieve a mean *P_Q_*_1_(*t*,*d*) of 63.7% and *P_Q_*_1_(*t*) of 64.3%, substantially above the average of the entire PDB (58.6% and 57.7%, respectively). These differences are statistically significant according to Welch's *t*‐test (*p* < 0.001) and are much more prominent than those reported in the R&K study [19]. This discrepancy most probably stems from the fact that in our study we used a relative measure that combines several quality metrics, and had 2.3 times more structural genomics deposits at our disposal and 6.1 times more structures overall.

When looking at the most popular journals, that is, those with more than 1000 structures (Table 3, gray rows), the top three spots are occupied by *Biochemical Journal*, *FEBS Journal*, and *Nature Chemical Biology*. At the other end of the spectrum, we have *EMBO Journal*, *Cell*, and *Nature Structural & Molecular Biology*, which were ranked last according to *P_Q_*_1_(*t*,*d*). It is worth noting that the latter three journals are the only journals that have average *P_Q_*_1_(*t*,*d*) below 50%. This means that, on average, at the time of deposition, the structures presented in these journals were already worse than over 50% of PDB structures of similar resolution. A similar ranking was obtained using *P_Q_*_1_(*t*) (Table S1), the main difference being that journals publishing structures at superior resolution, such *Chemistry* or *Acta Crystallographica D*, achieved much higher positions in the journal ranking. Table 3 and Tables S1–S3 also identify journals whose average *P_Q_*_1_(*t*,*d*), *P_Q_*_1_(*t*), $P_{Q1_{\min}}\left(t,d\right)$ , and $P_{Q1_{\min}}\left(t\right)$ are significantly different from the expected values of the entire PDB population.

It should be noted that the ranking presented in Table 3 takes into account over 45 years of structural data. This means that the ranking averages the entire lifespans of journals, which in their own individual history might have evolved over time. That is why in the following section we analyze how the ranking of the most popular journals has changed over the years.

### Quality of journals' structures over time

**Fig. 3:**
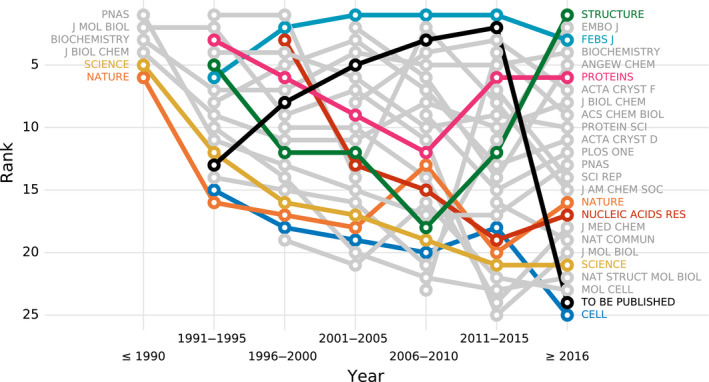
Journal ranking over time according to *P_Q_*_1_(*t*,*d*). The plot shows the journal's rank (*y*‐axis) in a given time period (*x*‐axis). The ranking includes 25 most popular journals, that is, journals with most structures, ranked based on structures deposited within 5‐year windows. A point appears only if a journal published at least 30 structures in a given 5‐year interval.

Owing to the fact that *P_Q_*_1_(*t*,*d*) assesses structures at the time of deposition, we also analyzed rankings of journals as a function of time. Figure 3 presents the ranking of 25 all‐time most popular journals in periods of 5 years. To minimize the effect of noise on the ranking, journals were assigned to a given 5‐year period only when they contained primary citations to at least 30 structures within that period.

As Fig. 3 shows only six of the 25 journals published at least 30 structures before 1991, however, these six journals were the primary reference for 482 out of 666 PDB deposits from this period. *Biochemistry* remains one of the top journals in terms of structure quality to date, *PNAS* and *J Biol Chem* are in the middle of the ranking, whereas *Nature*, *Science*, and *J Mol Biol* occupy the bottom half of the ranking. A journal that has steadily remained at the top of the ranking list for most of the years is *FEBS Journal*. Apart from *Biochemistry* and *FEBS Journal*, *Proteins* can also pride itself with a solid presence in the top 10 of the ranking throughout the years. It is worth noting that these three journals were also highly ranked in the study of B&R [18].

Disappointingly, the relatively poor ranks of highly reputable venues are not a new concern, but rather have been a steady trend for many years. It must be noted, however, that the overall structure quality of practically all 25 of the most popular journals has greatly improved in the last ten years, with *Science* and *Nature* noting the most positive trends (Fig. S5). Similar observations were made when the journals were ranked according to *P_Q_*_1_(*t*) (Figs S6 and S7).

A separate comment is required for the ‘venue’ *To be published*, most frequently found in PDB deposits. This category of PDB entries, omitted in the studies of B&R [18] and R&K [19], presents a very interesting pattern over the years. For several decades, unpublished structures (because staying in the ‘to be published’ state for several years in practice means ‘unpublished’) noted a steady upward trend, coming as far as the second place among the most popular venues between 2011 and 2015. The low position in the latest ranking period (2016–today) may stem from the fact that many of the recently deposited *To be published* structures from this time range still have a chance of being published and are under peer review. The retrospective pattern is that structures in the *To be published* category have higher *P_Q_*_1_(*t*,*d*) (61.4%) than structures that are presented in concrete journals (58.0%) (Welch's *t*‐test, *p* < 0.001). Moreover, 8543 out of 12 494 structural genomics structures remain unpublished even as structure notes, constituting over 66% and 33% of all unpublished structures in the 2005–2010 and 2011–2015 periods, respectively. With fewer structural genomics depositions in the last four years (only 15% of all unpublished structures for this period), the *To be published* category currently has a lower ratio of high‐quality structures. This, combined with the observed constant improvement of published structures, may further contribute to the drop of the *To be published* category in the ranking.

### Retrospective comparison with the results of Brown and Ramaswamy

The journal rankings presented in this work were inspired by the study of Brown and Ramaswamy (B&R) [18]. Although the methodologies used in these two analyses are different (most notably because of incorporation of nucleic acids and the use of data imputation in the present work), it is worth verifying how the two approaches compare, and what has changed since the original B&R study. To help answer these questions, Table 4 presents the journal ranking reported by B&R in 2007 together with two lists of the same journals ranked according to *P_Q_*_1_(*t*,*d*): based on PDB deposits available in 2007 and based on all currently available data.

**Table 4:** Comparison of journal ranking by Brown and Ramaswamy [18] with rankings of the same journals created using *P_Q_*_1_(*t*,*d*). Numbers of structures considered from a given journal are shown in parentheses. The top three journals according to B&R are highlighted in green, and the bottom three journals are highlighted in red

B&R ranking [18] (year < 2007)	Ranking according to *P_Q_*_1_(*t*,*d*) (year < 2007)	Ranking according to *P_Q_*_1_(*t*,*d*) (current)
*FEBS J*^a^ (159)	*J Biol Inorg Chem* (114)	*Biochem J* ^a^ (1033)
*Protein Eng Des Sel* (96)	*FEBS J*^a^ (356)	*FEBS J*^a^ (1539)
*Biochemistry*^a^ (3346)	*Protein Eng Des Sel* (173)	*J Biol Inorg Chem* (265)
*Cell Chem Biol* (154)	*Acta Cryst D* ^a^ (1766)	*FEBS Lett* ^a^ (814)
*Proteins* (398)	*Biochem J* (165)	*J Am Chem Soc* ^a^ (2369)
*J Mol Biol* (3855)	*FEBS Lett* (232)	*Protein Eng Des Sel* (294)
*Acta Cryst D* (1074)	*Cell Chem Biol* (204)	*Cell Chem Biol* (902)
*Protein Sci* (771)	*Protein Sci* ^a^ (1104)	*Acta Cryst D* ^a^ (4952)
*Bioorg Med Chem Lett* (195)	*Biochemistry*^a^ (4564)	*Biochemistry*^a^ (8896)
*J Struct Biol* (83)	*J Mol Biol* ^a^ (5497)	*Nucleic Acids Res* (2127)
*Biophys J* (71)	*Biophys J* (95)	*Structure* (5348)
*J Biol Inorg Chem* (81)	*Proteins* (993)	*Protein Sci* (2235)
*Biochem J* (67)	*J Am Chem Soc* (459)	*J Med Chem* (5525)
*J Biol Chem* (2849)	*Biochem Biophys Res Commun* (167)	*J Virol* (957)
*J Am Chem Soc* (324)	*Nucleic Acids Res* (288)	*J Biol Chem* (11055)
*Structure* (1412)	*J Bacteriol* (141)	*J Struct Biol* (1038)
*FEBS Lett* (173)	*Bioorg Med Chem* (59)	*J Bacteriol* (371)
*J Bacteriol* (111)	*J Biol Chem* (4090)	*Bioorg Med Chem* (659)
*Bioorg Med Chem* (53)	*J Struct Biol* (117)	*Proteins* (1999)
*J Med Chem* (450)	*J Med Chem* (605)	*Biochem Biophys Res Commun* (976)
*Nat Struct Mol Biol* (768)	*Structure* (2017)	*PNAS* (7376)
*PNAS* (1324)	*PNAS* ^a^ (1839)	*Biophys J* (199)
*J Virol* (86)	*J Virol* (141)	*J Mol Biol* ^a^ (9507)
*Biochem Biophys Res Commun* (103)	*Science*^a^ (712)	*Nature* ^a^ (3060)
*EMBO J* ^a^ (768)	*EMBO J* ^a^ (1135)	*Science*^a^ (1949)
*Nucleic Acids Res* (199)	*Nat Struct Mol Biol* ^a^ (1342)	*Mol Cell*^a^ (1599)
*Nature* ^a^ (807)	*Nature* ^a^ (976)	*Bioorg Med Chem Lett* ^a^ (1590)
*Mol Cell*^a^ (422)	*Bioorg Med Chem Lett* ^a^ (323)	*Nat Struct Mol Biol* ^a^ (2915)
*Science*^a^ (571)	*Cell*^a^ (647)	*Cell*^a^ (1563)
*Cell*^a^ (488)	*Mol Cell*^a^ (571)	*EMBO J* ^a^ (1910)

^a^ Journals whose quality was determined to be significantly different from the average quality of structures the entire PDB, at significance level α = 0.001.

It can be noticed that the rankings bear several similarities, although they are not identical. Journals that were at the top of the B&R ranking generally remain highly ranked according to *P_Q_*_1_(*t*,*d*). Similarly, the bottom regions of the rankings are occupied by the same group of journals. However, there are some notable differences. For example, *Bioorg Med Chem Lett* is ranked 19 places lower according to *P_Q_*_1_(*t*,*d*), whereas *J Biol Inorg Chem*, *FEBS Lett*, and *Nucleic Acids Res* are ranked 11 places higher. These differences may be the result of the number of structures taken into account by each ranking. Compared to the time of the B&R study, significantly more precomputed quality metrics are now available, even for older PDB deposits. Moreover, the methodology proposed in this work imputes missing values, allowing for inclusion of 12.7% additional structures. As a result, the rankings based on *P_Q_*_1_(*t*,*d*) were compiled using much more data, occasionally changing a journal's rank substantially.

### Correlation between structure quality and journal impact

The low ranking of high‐impact journals in the current study raises the question of whether structure quality is negatively correlated with journal impact. The study of B&R [18] strongly suggested that this was the case, whereas the slightly more recent work of R&K [19] showed that the differences in structure quality between high‐impact and other venues were relatively small. However, both studies manually categorized journals as high‐ or low‐impact venues rather than investigating actual impact metrics for a large set of journal titles.

In this study, we decided to measure journal impact quantitatively and correlate it with our quantitative measure of structure quality. For this purpose, we used two metrics: impact per publication (IPP) and source normalized impact per paper (SNIP) [30]. IPP is calculated the same way as 3‐year impact factor (IF3) but using only publications that are classified as articles, conference papers, or reviews in Scopus. SNIP is a modification of IPP that corrects for differences in citation practices between scientific fields [30]. Both journal metrics have 20 years (1999–2018) of publicly available statistics and are based on the same source data.

**Fig. 4:**
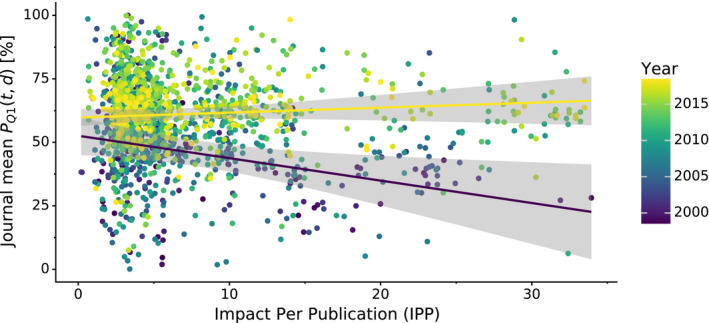
Scatterplot of mean journal *P_Q_*_1_(*t*,*d*) and the journal's impact over time. Variation in mean journal *P_Q_*_1_(*t*,*d*) (*y*‐axis) in a given year (color) plotted against the journals IPP. IPP uses the same formula as the 3‐year impact factor, but is based on publicly available Scopus data. The two regression lines show linear trends for 1999 (indigo) and 2018 (yellow) along with 95% confidence intervals (gray areas).

**Fig. 5:**
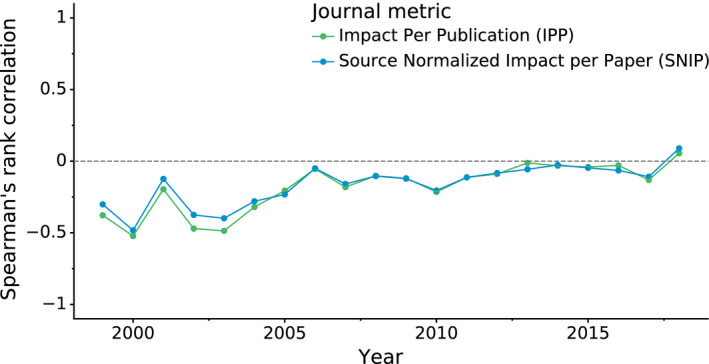
Correlation between structure quality and journal impact. The plot shows Spearman's rank correlation (*y*‐axis) over time (*x*‐axis) between structure quality measured by *P_Q_*_1_(*t*,*d*) and journal impact measured using the IPP and SNIP metrics. IPP uses the same formula as the 3‐year impact factor but is based on Scopus data, whereas SNIP additionally takes into account the scientific field.

Figure 4 shows the relation between *P_Q_*_1_(*t*,*d*) and the journal impact over time (separate plots for each year are presented in Fig. S8). It is evident that structure quality has substantially improved over the last decade and that the negative correlation between journal impact and the quality of structural models presented therein seems to disappear as time progresses. This observation is confirmed when the relation between journal impact (IPP, SNIP) and structure quality (*P_Q_*_1_(*t*,*d*)) is gauged using Spearman's rank correlation coefficient. Figure 5 shows that even though structure quality and journal impact were indeed negatively correlated 20 years ago, currently there is no correlation between these two criteria.

Figure 4 also shows a very interesting situation in the low‐IF range, namely that low‐IF journals publish just about anything: the most fantastic work as well as structures beneath contempt. On the other hand, medium‐IF journals used to be primary citations of mostly poor structures in the past. At present, however, they are doing a much better job, publishing mostly better‐than‐average structures.

## Discussion

Our analysis confirms recent reports that the quality of crystallographic macromolecular structures has improved over the last years [16]. However, we also found out that at the time of the B&R analysis the quality of PDB structures temporarily stopped improving and that this is most likely why B&R did not report any correlation between quality and time [18]. In addition to confirming earlier findings, by using a data imputation algorithm we were able to put into context the quality of structural models going back in time as far as 1972. As convincingly illustrated by Figs 1 and 2, the quality of PDB structures had rapidly improved over the first two decades of the database.

The ability to analyze quality over time using the proposed *P_Q_*_1_(*t*,*d*) measure (Fig. 3) shows that there is tight competition among journals as their number increases. Quite interestingly, it is also evident that the PDB treasures many good quality structures that do not have primary citations. The fact that a structure remains *To be published* indicates that it is getting more and more difficult to publish papers based solely on crystallographic results, even if they are of high quality. Indeed, our study shows that structures without primary citations are on average of higher quality than structures published in many popular journals. Therefore, although many structures do not have any accompanying journal publications, they present a substantial value in their own right. As each PDB deposit has its own digital object identifier (DOI), citation of structures should be acknowledged not only by PDB IDs but also by DOIs. Full implementation of this mechanism would allow for easy estimation of the impact of *To be published* structures.

The proposed *P_Q_*_1_(*t*,*d*) and *P_Q_*_1_(*t*) measures manifest the overall attitude of authors toward the quality of the PDB: Each next deposited structure should be better than the average previous deposit. Each new quality metric [20], visualization technique [31], set of restraints [32], validation algorithm [33], hardware improvement [34], or software update [35] makes it easier to produce good quality structures and to avoid simple mistakes. In an effort to promote constant improvement of overall PDB quality, it would be a desirable ideal to expect that newly added models are above the current average. However, such a recommendation should be applied judiciously as each case is different and should be always judged in a context‐dependent manner. It is gratifying to see that almost all journals publish structures that are, on average, better than most of the previous ones while those that are not at that level yet seem to be heading in the right direction.

## Methods

### Data collection and cleaning

To provide a comprehensive analysis of structure quality over time, we examined all X‐ray structures available in the PDB that included 141 154 deposits between 1972 and 2019. The data were downloaded by performing an SQL query on PDBj [36, 37] as of December 10, 2019.

In order to perform an analysis of structure quality in correlation with the primary citations, journal names had to be extracted from PDB files, cleaned, and unified. Initially, the dataset contained 1342 unique values describing the primary citation journal. After eliminating typos, unifying punctuation and ISSNs, and taking into account that some journals have changed their titles over time, the number of unique journal names dropped down to 800.

Bibliometric indicators of journals (IPP, SNIP) were downloaded from the Leiden University CWTS website (https://www.journalindicators.com/) and joined with the PDB data using ISSNs. Both indicators were calculated based on the Scopus bibliographic database produced by Elsevier.

### Missing data imputation

To fill in missing data, three approaches were tested: filling missing values with the metric's mean value, the metric's median, and using the multiple imputation by chained equations algorithm (MICE) [22, 23] with Bayesian ridge regression [24] as the predictor.

To see how well each of the three methods performed, the nonmissing (i.e., complete) portion of the PDB data was used as the basis for creating a test set. We randomly introduced missing values to the complete portion of the data in the same proportions as those present in the actual dataset. As a result, the test dataset had the same proportion of deposits with at least one missing value and the same percentage of missing values per metric as the original (full) dataset. Next, these randomly introduced missing values were imputed and compared against the values originally present in the dataset. To quantify the imputation error, we used the MAD, MAE, and RMSE error estimation methods [38]. The procedure was repeated 100 times with different subsets of values randomly eliminated from the complete dataset in each run. Imputed missing values were clipped when they were outside the range of possible values of a given metric.

### Principal component analysis

The PCA required to calculate *Q*1_p_ was performed as described by Shao *et al.* [16]. The PCA was performed on three quality metrics: Clashscore, Ramachandran outliers, and Rotamer outliers. Since Ramachandran outliers and Rotamer outliers are meaningful only for proteins, the PCA was performed for protein structures only. In the assessment of the quality of nucleic acid structures, the PCA step was not needed, as Clashscore was the only geometry‐related quality index.

Upon visual inspection of the metrics' values (Fig. S9), structures were marked as outliers and removed when the following criteria were reached: Rotamer outliers > 50% or Ramachandran outliers > 45% or Clashscore > 250. In total, 16 structures were marked as outliers: 1C4D, 1DH3, 1G3X, 1HDS, 1HKG, 1HPB, 1PYP, 1SM1, 2ABX, 2GN5, 2OGM, 2Y3J, 3ZS2, 4BM5, 4HIV, and 5M2K. These structures were temporarily removed prior to PCA to decrease the effect of outlying values on the principal components, but they were not removed from the quality analysis. After removal of outstanding outliers, the input data for PCA were standardized by setting the mean to be 0 and standard deviation to 1. Running PCA on the standardized data resulted in three principal components: PC1, PC2, and PC3, explaining 78%, 14%, and 8% variance, respectively. The coefficients of PC1 were 0.60, 0.58, and 0.56, indicating nearly equal contribution of Clashscore, Ramachandran outliers, and Rotamer outliers. The explained variance of each principal component and the coefficients of PC1 were practically identical to those reported by Shao *et al.* [16].

As noted by one of the reviewers, the PC1 coefficients (0.60, 0.58, 0.56) are almost identical and roughly equal $\frac{1}{\sqrt{3}}$ , making the respective weights of these contributions near equal for all three of them. This means that approximately the *Q*1_p_ measure could be presented as follows:

(5) $$Q1_{p}=\frac{P_{R_{\text{free}}}+P_{\%\text{RSRZ}}+\frac{1}{\sqrt{3}}\left(P_{\text{Clashscore}}+P_{\text{Ramachandran}}+P_{\text{Rotamers}}\right)}{3}.$$

The above formula provides a simple metric that can be used without performing PCA. However, this approximate formula assumes that the relations between Clashscore, Ramachandran outliers, and Rotamer outliers are fixed and will not change. For this reason, we chose to use the exact formula (1) as proposed by Shao *et al.* [16]. Nevertheless, the approximate formula (5) may be considered a simpler solution for less technical studies.

### Computation

Data were extracted directly from PDBj using its SQL interface. All computations were performed with python 3.7 using the scipy [39] and scikit‐learn [40] libraries. The SQL query used, the resulting dataset, and fully reproducible analysis scripts in the form of a Jupyter notebook are available at https://github.com/dabrze/pdb_structure_quality.

## Conflict of interest

The authors declare no conflict of interest.

## Author contributions

MJ conceived this study, and coordinated the project and manuscript preparation. DB proposed the ranking measures, performed the experiments, and drafted the manuscript. ZD provided most of the initial data and participated in manuscript preparation. WM participated in data analysis and manuscript preparation.

## Supporting information


**Fig. S1.** Histograms of quality metric values of structures found in the PDB.
**Fig. S2.** *P_Q1_* analysis without imputed values.
**Fig. S3.** *P_Q1min_* analysis (minimum approach).
**Fig. S4.** Comparison of *P_Q1min_(t,d)* of protein and nucleic acid structures over time.
**Fig. S5.** Average *P_Q1_(t,d)* of popular journals for each year.
**Fig. S6.** Journal ranking over time according to *P_Q1_(t).*
**Fig. S7.** Journal quality over time according to *P_Q1_(t).*
**Fig. S8.** Scatterplots of mean journal *P_Q1_(t,d)* and the journal's impact. Scatterplots of mean journal *P_Q1_(t,d)* and the journal's impact.
**Fig. S9.** Scatterplots of the values of Clashscore, Ramachandran outliers, and Rotamer outliers found in the PDB.
**Table S1.** All‐time journal ranking according to *P_Q1_(t)*.
**Table S2.** All‐time journal ranking according to *P_Q1min_(t,d)*.
**Table S3.** All‐time journal ranking according to *P_Q1min_(t)*.

## Data Availability

Supplementary tables and figures are available at figshare.com: 10.6084/m9.figshare.11366222. Datasets and reproducible experimental scripts are available at GitHub: https://github.com/dabrze/pdb_structure_quality.
