# Supplementary material for: On the evolution of the quality of macromolecular models in the PDB
Source: FEBS J. 2020 Apr 20;287(13):2685–98. doi: 10.1111/febs.15314 (PMC7340579; doi:10.1111/febs.15314)
Supplement: Supplementary file 1 — Fig. S1. Histograms of quality metric values of structures found in the PDB. Fig. S2. PQ1 analysis without imputed values. Fig. S3. PQ1min analysis (minimum approach). Fig. S4. Comparison of PQ1min(t,d) of protein and nucleic acid structures over time. Fig. S5. Average PQ1(t,d) of popular journals for each year. Fig. S6. Journal ranking over time according to PQ1(t). Fig. S7. Journal quality over time according to PQ1(t). Fig. S8. Scatterplots of mean journal PQ1(t,d) and the journal's impact. Scatterplots of mean journal PQ1(t,d) and the journal's impact. Fig. S9. Scatterplots of the values of Clashscore, Ramachandran outliers, and Rotamer outliers found in the PDB. Table S1. All‐time journal ranking according to PQ1(t). Table S2. All‐time journal ranking according to PQ1min(t,d). Table S3. All‐time journal ranking according to PQ1min(t). [file FEBS-287-2685-s001.zip › febs15314-sup-0001-Supinfo.pdf]

FEBS PRESS
science publishing by scientists

WILEY

The FEBS Journal

www.febsjournal.org

On the evolution of the quality of macromolecular models in the PDB

Dariusz Brzezinski, Zbigniew Dauter, Wladek Minor and Mariusz Jaskolski

DOI: 10.1111/febs.15314

On the evolution of the quality of macromolecular models in the PDB
Supplementary Materials

Dariusz Brzezinski[1,2,3,4], Zbigniew Dauter[5], Wladek Minor[4] and Mariusz Jaskolski[1,6]*

[1]Center for Biocrystallographic Research, Institute of Bioorganic Chemistry, Polish Academy of Sciences, Poznan, 61-704, Poland; [2]Institute of Computing Science, Poznan University of Technology, Poznan, 60-965, Poland; [3]Center for Artificial Intelligence and Machine Learning, Poznan University of Technology, Poznan, 60-965, Poland; [4]Department of Molecular Physiology and Biological Physics, University of Virginia, Charlottesville, USA; [5]Synchrotron Radiation Research Section, Macromolecular Crystallography Laboratory, National Cancer Institute, Argonne National Laboratory, Argonne, USA; [6]Department of Crystallography, Faculty of Chemistry, A. Mickiewicz University, Poznan, 61-614, Poland;

*Correspondence e-mail: mariuszj@amu.edu.pl

Table S1. All-time journal ranking according to $P_{Q1}(t)$. The ranking includes all the journals that had at least 100 primary citations of structures in the PDB. $P_{Q1}(t)$ higher than 50% means that the structures published in a given journal were, on average, better than 50% of structures present in the PDB at the time of deposition (in this case regardless of resolution). Journals with more than 1000 structures are highlighted in gray.

* Denotes journals that have average $P_{Q1}(t)$ significantly different than the expected value in the population, according to Welch's t-test with Bonferroni correction at the significance level α=0.001. Mean denotes the arithmetic mean, G-mean denotes the geometric mean (log-average), V-mean denotes the mean in $Å^{-1/3}$.

Rank	Journal	Mean $P_{Q1}(t)$ [%]	Mean resolution [Å]	G-mean resolution [Å]	V-mean resolution [Å]	Structure count
1	TUBERCULOSIS (EDINB)*	84.81	2.02	2.00	1.92	132
2	CHEMISTRY*	72.34	1.82	1.77	1.62	242
3	CHEM COMMUN*	71.88	1.81	1.75	1.59	280
4	CHEMBIOCHEM*	71.84	1.92	1.87	1.71	527
5	ACS INFECT DIS*	71.69	1.95	1.90	1.78	153
6	EUR J MED CHEM*	71.39	2.03	1.97	1.81	418
7	ACS CATAL*	71.33	1.94	1.88	1.70	241
8	ORG BIOMOL CHEM*	71.22	1.88	1.82	1.64	167
9	J INORG BIOCHEM*	71.11	1.78	1.73	1.57	171
10	IUCRJ*	70.62	1.95	1.88	1.69	281
11	J SYNCHROTRON RADIAT*	70.24	1.79	1.73	1.56	147
12	ACS OMEGA*	69.77	1.81	1.77	1.64	102
13	J BIOL INORG CHEM*	68.05	1.88	1.82	1.64	265
14	CHEM SCI*	67.78	1.87	1.83	1.68	268
15	J COMPUT AIDED MOL DES	67.57	1.88	1.86	1.77	115
16	ANGEW CHEM*	67.45	1.92	1.85	1.65	1,065
17	NAT CHEM*	67.45	1.95	1.85	1.64	173
18	FEBS J*	66.24	2.02	1.97	1.81	1,539
19	APPL ENVIRON MICROBIOL	66.00	2.01	1.96	1.83	112
20	ANTIMICROB AGENTS CHEMOTHER*	65.64	1.96	1.88	1.65	309
21	ARCH BIOCHEM BIOPHYS*	65.33	2.08	2.03	1.89	276
22	GLYCOBIOLOGY	65.13	1.96	1.90	1.74	188
23	ACS CHEM BIOL*	64.70	2.04	1.98	1.83	1,104

24	CHEMMEDCHEM*	64.65	1.94	1.88	1.69	556
25	BIOCHEM J*	64.32	2.11	2.06	1.89	1,033
26	INT J MOL SCI	63.83	2.12	2.04	1.81	103
27	ACTA CRYST F*	63.71	2.09	2.02	1.81	1,466
28	ACTA CRYST D*	63.61	1.99	1.91	1.70	4,952
29	J AM CHEM SOC*	63.37	2.00	1.93	1.74	2,369
30	PROTEIN ENG DES SEL	63.05	2.05	2.00	1.84	294
31	TO BE PUBLISHED*	62.75	2.03	1.98	1.81	22,421
32	BIOPHYS J	62.49	1.92	1.85	1.66	199
33	MBIO	62.04	2.24	2.15	1.91	169
34	SCI REP*	61.27	2.16	2.09	1.88	1,847
35	NAT CHEM BIOL	61.12	2.19	2.12	1.92	1,013
36	FEBS LETT	61.10	2.10	2.04	1.85	814
37	BIOCHEMISTRY*	60.68	2.05	2.00	1.84	8,896
38	PLOS PATHOG	60.66	2.25	2.17	1.96	656
39	CELL CHEM BIOL	60.51	2.08	2.03	1.88	902
40	J BIOCHEM	60.30	2.05	2.01	1.88	279
41	PLOS ONE	60.05	2.15	2.09	1.92	2,057
42	NAT COMMUN*	60.04	2.21	2.11	1.86	3,538
43	FASEB J	60.01	2.03	1.98	1.86	161
44	BMC STRUCT BIOL	59.69	2.08	2.02	1.84	228
45	BIOCHIMIE	59.58	2.07	2.02	1.81	128
46	PROTEIN SCI	59.57	2.07	2.02	1.85	2,235
47	ACS MED CHEM LETT	59.41	2.12	2.06	1.88	1,062
48	J MED CHEM	58.68	2.07	2.02	1.86	5,525
49	BIOCHIM BIOPHYS ACTA	58.15	2.09	2.04	1.87	600
50	PROTEINS	57.97	2.07	2.01	1.84	1,999
51	BIOORG MED CHEM	57.87	2.10	2.04	1.88	659
52	MOL MICROBIOL	57.66	2.16	2.09	1.87	412
53	J BIOL CHEM	57.41	2.12	2.06	1.89	11,055
54	NAT MICROBIOL	56.88	2.32	2.25	2.04	111
55	J STRUCT BIOL	56.58	2.15	2.08	1.90	1,038
56	INT J BIOL MACROMOL	56.48	2.05	2.00	1.80	168
57	J STRUCT FUNCT GENOM	55.69	2.07	2.03	1.92	168
58	STRUCTURE*	55.54	2.20	2.11	1.86	5,348
59	J MOL BIOL*	55.23	2.13	2.06	1.88	9,507
60	VIROLOGY	54.62	2.44	2.37	2.18	126
61	BIOCHEM BIOPHYS RES COMMUN	54.29	2.18	2.11	1.92	976
62	MABS	54.14	2.35	2.29	2.14	115
63	PLANT CELL	53.65	2.21	2.17	2.05	138
64	J BACTERIOL	53.63	2.22	2.16	2.01	371
65	NUCLEIC ACIDS RES*	53.31	2.28	2.21	1.98	2,127
66	CELL HOST MICROBE	53.10	2.50	2.43	2.20	107
67	J VIROL*	52.22	2.34	2.26	2.06	957
68	PNAS*	52.17	2.27	2.19	1.94	7,376
69	SCI ADV	51.69	2.33	2.22	1.85	182
70	MOL PHARMACOL	51.48	2.35	2.27	2.02	129
71	RNA	51.48	2.44	2.32	1.97	245
72	PLOS BIOL	51.12	2.35	2.25	2.01	336
73	J EXP MED	50.54	2.34	2.28	2.08	121
74	ELIFE*	49.25	2.41	2.30	2.03	869
75	J IMMUNOL*	49.01	2.26	2.19	2.02	296
76	PROTEIN CELL	48.77	2.26	2.21	2.04	178
77	BIOORG MED CHEM LETT*	47.44	2.19	2.16	2.05	1,590
78	EMBO REP*	47.25	2.35	2.28	2.09	211
79	CELL REP*	46.89	2.52	2.42	2.16	399
80	GENES DEV*	46.62	2.41	2.33	2.11	279
81	CELL RES*	45.36	2.42	2.36	2.20	189
82	SCIENCE*	44.98	2.50	2.39	2.10	1,949
83	NATURE*	44.77	2.52	2.42	2.12	3,060
84	NEURON*	44.65	2.63	2.41	2.08	149
85	NAT STRUCT MOL BIOL*	44.10	2.40	2.31	2.07	2,915
86	COMMUN BIOL*	43.81	2.39	2.31	2.00	104
87	EMBO J*	43.63	2.37	2.30	2.10	1,910
88	IMMUNITY*	43.18	2.44	2.37	2.18	265
89	MOL CELL*	42.69	2.45	2.37	2.14	1,599
90	NAT IMMUNOL*	42.15	2.52	2.45	2.22	119
91	CELL*	40.07	2.54	2.45	2.20	1,563

Table S2. All-time journal ranking according to *$P_{Q1min}(t,d)$*. The ranking includes all the journals that had at least 100 primary citations of structures in the PDB. $P_{Q1min}(t,d)$ higher than 50% means that the structures published in a given journal were, on average, better than 50% of structures present in the PDB at the time of deposition. Journals with more than 1000 structures are highlighted in gray.

* Denotes journals that have average $P_{Q1min}(t,d)$ significantly different than the expected value in the population, according to Welch's t-test with Bonferroni correction at the significance level α=0.001. Mean denotes the arithmetic mean, G-mean denotes the geometric mean (log-average), V-mean denotes the mean in $Å^{-1/3}$.

Rank	Journal	Mean $P_{Q1min}(t,d)$ [%]	Mean resolution [Å]	G-mean resolution [Å]	V-mean resolution [Å]	Structure count
1	TUBERCULOSIS (EDINB)*	82.20	2.02	2.00	1.92	132
2	CELL HOST MICROBE	67.84	2.50	2.43	2.20	107
3	EUR J MED CHEM*	67.32	2.03	1.97	1.81	418
4	ACS CATAL*	67.12	1.94	1.88	1.70	241
5	ORG BIOMOL CHEM	66.21	1.88	1.82	1.64	167
6	CHEMBIOCHEM*	66.02	1.92	1.87	1.71	527
7	ACS INFECT DIS	64.24	1.95	1.90	1.78	153
8	ARCH BIOCHEM BIOPHYS	64.14	2.08	2.03	1.89	276
9	CHEM COMMUN	63.92	1.81	1.75	1.59	280
10	MBIO	63.76	2.24	2.15	1.91	169
11	IUCRJ	63.72	1.95	1.88	1.69	281
12	BIOCHEM J*	63.32	2.11	2.06	1.89	1,033
13	INT J MOL SCI	63.24	2.12	2.04	1.81	103
14	J BIOL INORG CHEM	63.12	1.88	1.82	1.64	265
15	PLOS PATHOG*	62.66	2.25	2.17	1.96	656
16	NAT CHEM BIOL*	62.30	2.19	2.12	1.92	1,013
17	FEBS J*	62.28	2.02	1.97	1.81	1,539
18	APPL ENVIRON MICROBIOL	62.13	2.01	1.96	1.83	112
19	RNA	61.14	2.44	2.32	1.97	245
20	ACTA CRYST F*	61.10	2.09	2.02	1.81	1,466
21	NAT MICROBIOL	60.95	2.32	2.25	2.04	111
22	CHEMISTRY	60.79	1.82	1.77	1.62	242
23	SCI REP*	60.75	2.16	2.09	1.88	1,847
24	ANGEW CHEM	60.28	1.92	1.85	1.64	1,065
25	J EXP MED	60.22	2.34	2.28	2.08	121
26	BIOCHIMIE	60.15	2.07	2.02	1.81	128
27	MOL PHARMACOL	60.03	2.35	2.27	2.02	129
28	J SYNCHROTRON RADIAT	59.89	1.79	1.73	1.56	147
29	J INORG BIOCHEM	59.58	1.78	1.73	1.57	171
30	ANTIMICROB AGENTS CHEMOTHER	59.38	1.96	1.88	1.65	309
31	NAT COMMUN*	59.36	2.21	2.11	1.86	3,538
32	FEBS LETT	59.34	2.10	2.04	1.85	814
33	ACS CHEM BIOL	59.29	2.04	1.98	1.83	1,104
34	PLOS ONE	59.25	2.15	2.09	1.92	2,057
35	PROTEIN ENG DES SEL	59.00	2.05	2.00	1.84	294
36	GLYCOBIOLOGY	58.97	1.96	1.90	1.74	188
37	J VIROL	58.63	2.34	2.26	2.06	957
38	CELL CHEM BIOL	58.61	2.08	2.03	1.88	902
39	MABS	58.48	2.35	2.29	2.14	115
40	J AM CHEM SOC	58.36	2.00	1.93	1.74	2,369
41	NAT CHEM	58.34	1.95	1.85	1.65	173
42	STRUCTURE	58.27	2.20	2.11	1.86	5,348
43	BIOCHEMISTRY	58.26	2.05	2.00	1.84	8,896
44	VIROLOGY	58.08	2.44	2.37	2.18	126
45	CELL REP	58.02	2.52	2.42	2.16	399
46	**TO BE PUBLISHED**	57.99	2.03	1.98	1.81	22,421
47	PLANT CELL	57.97	2.21	2.17	2.05	138
48	FASEB J	57.71	2.03	1.98	1.86	161
49	ACTA CRYST D	57.66	1.99	1.91	1.70	4,952
50	MOL MICROBIOL	57.64	2.16	2.09	1.87	412
51	ACS MED CHEM LETT	57.39	2.12	2.06	1.88	1,062
52	BMC STRUCT BIOL	57.30	2.08	2.02	1.84	228
53	J COMPUT AIDED MOL DES	56.93	1.88	1.86	1.77	115
54	NUCLEIC ACIDS RES	56.81	2.28	2.21	1.98	2,127
55	CHEMMEDCHEM	56.77	1.94	1.88	1.69	556
56	ACS OMEGA	56.65	1.81	1.77	1.64	102
57	BIOCHIM BIOPHYS ACTA	56.63	2.09	2.04	1.87	600
58	PROTEIN SCI	56.54	2.07	2.02	1.85	2,235
59	J BIOL CHEM	56.37	2.12	2.06	1.89	11,055
60	J MED CHEM	56.18	2.07	2.02	1.86	5,525
61	J BIOCHEM	56.06	2.05	2.01	1.88	279

62	PROTEINS	55.96	2.07	2.01	1.84	1,999
63	NATURE	55.78	2.52	2.42	2.12	3,060
64	CELL RES	55.67	2.42	2.36	2.20	189
65	J BACTERIOL	55.64	2.22	2.16	2.01	371
66	BIOCHEM BIOPHYS RES COMMUN	55.52	2.18	2.11	1.92	976
67	NAT IMMUNOL	55.46	2.52	2.45	2.22	119
68	J MOL BIOL*	55.21	2.13	2.06	1.88	9,507
69	PNAS*	55.10	2.27	2.19	1.94	7,376
70	J STRUCT BIOL	55.09	2.15	2.08	1.90	1,038
71	CHEM SCI	55.02	1.87	1.83	1.68	268
72	BIOORG MED CHEM	54.85	2.10	2.04	1.88	659
73	PLOS BIOL	54.37	2.35	2.25	2.01	336
74	SCI ADV	54.34	2.33	2.22	1.85	182
75	PROTEIN CELL	54.17	2.26	2.21	2.04	178
76	J IMMUNOL	54.06	2.26	2.19	2.02	296
77	COMMUN BIOL	54.02	2.39	2.31	2.00	104
78	GENES DEV	53.88	2.41	2.33	2.11	279
79	ELIFE	53.83	2.41	2.30	2.03	869
80	SCIENCE*	53.67	2.50	2.39	2.10	1,949
81	IMMUNITY	53.20	2.44	2.37	2.18	265
82	EMBO REP	53.11	2.35	2.28	2.09	211
83	NEURON	52.38	2.63	2.41	2.08	149
84	NAT STRUCT MOL BIOL*	52.33	2.40	2.31	2.07	2,915
85	INT J BIOL MACROMOL	52.32	2.05	2.00	1.80	168
86	EMBO J*	52.20	2.37	2.30	2.10	1,910
87	MOL CELL*	51.94	2.45	2.37	2.14	1,599
88	CELL*	51.90	2.54	2.45	2.20	1,563
89	BIOPHYS J	50.44	1.92	1.85	1.66	199
90	BIOORG MED CHEM LETT*	50.01	2.19	2.16	2.05	1,590
91	J STRUCT FUNCT GENOM	49.35	2.07	2.03	1.92	168

Table S3. All-time journal ranking according to $P_{Q1min}(t)$. The ranking includes all the journals that had at least 100 primary citations of structures in the PDB. $P_{Q1min}(t)$ higher than 50% means that the structures published in a given journal were, on average, better than 50% of structures present in the PDB at the time of deposition (in this case regardless of resolution). Journals with more than 1000 structures are highlighted in gray.

* Denotes journals that have average $P_{Q1min}(t)$ significantly different than the expected value in the population, according to Welch's t-test with Bonferroni correction at the significance level α=0.001. Mean denotes the arithmetic mean, G-mean denotes the geometric mean (log-average), V-mean denotes the mean in $Å^{-1/3}$.

Rank	Journal	Mean $P_{Q1min}(t)$ [%]	Mean resolution [Å]	G-mean resolution [Å]	V-mean resolution [Å]	Structure count
1	TUBERCULOSIS (EDINB)*	81.93	2.02	2.00	1.92	132
2	ORG BIOMOL CHEM*	70.36	1.88	1.82	1.64	167
3	CHEM COMMUN*	70.06	1.81	1.75	1.59	280
4	CHEMBIOCHEM*	69.79	1.92	1.87	1.71	527
5	ACS CATAL*	69.56	1.94	1.88	1.70	241
6	EUR J MED CHEM*	68.11	2.03	1.97	1.81	418
7	ACS INFECT DIS*	67.92	1.95	1.90	1.78	153
8	J INORG BIOCHEM*	67.67	1.78	1.73	1.57	171
9	J BIOL INORG CHEM*	67.23	1.88	1.82	1.64	265
10	CHEMISTRY*	67.10	1.82	1.77	1.62	242
11	J SYNCHROTRON RADIAT*	66.90	1.79	1.73	1.56	147
12	IUCRJ*	66.32	1.95	1.88	1.69	281
13	APPL ENVIRON MICROBIOL	64.87	2.01	1.96	1.83	112
14	ANGEW CHEM*	64.12	1.92	1.85	1.64	1,065
15	J COMPUT AIDED MOL DES	64.01	1.88	1.86	1.77	115
16	ARCH BIOCHEM BIOPHYS*	63.73	2.08	2.03	1.89	276
17	ACS OMEGA	63.52	1.81	1.77	1.64	102
18	FEBS J*	63.40	2.02	1.97	1.81	1,539
19	NAT CHEM	62.95	1.95	1.85	1.65	173
20	ANTIMICROB AGENTS CHEMOTHER	62.47	1.96	1.88	1.65	309
21	BIOCHEM J*	62.19	2.11	2.06	1.89	1,033
22	PROTEIN ENG DES SEL	60.94	2.05	2.00	1.84	294
23	ACTA CRYST F*	60.83	2.09	2.02	1.81	1,466
24	GLYCOBIOLOGY	60.66	1.96	1.90	1.74	188
25	BIOCHIMIE	60.51	2.07	2.02	1.81	128
26	J AM CHEM SOC*	60.39	2.00	1.93	1.74	2,369
27	ACS CHEM BIOL*	60.35	2.04	1.98	1.83	1,104
28	CHEM SCI	60.20	1.87	1.83	1.68	268
29	ACTA CRYST D*	60.15	1.99	1.91	1.70	4,952
30	INT J MOL SCI	60.13	2.12	2.04	1.81	103
31	BIOCHEMISTRY*	59.55	2.05	2.00	1.84	8,896
32	CELL CHEM BIOL	59.40	2.08	2.03	1.88	902
33	FASEB J	59.35	2.03	1.98	1.86	161
34	**TO BE PUBLISHED***	59.33	2.03	1.98	1.81	22,421
35	NAT CHEM BIOL	59.26	2.19	2.12	1.92	1,013
36	FEBS LETT	59.04	2.10	2.04	1.85	814
37	CHEMMEDCHEM	58.95	1.94	1.88	1.69	556
38	SCI REP	58.49	2.16	2.09	1.88	1,847
39	MBIO	58.47	2.24	2.15	1.91	169
40	PLOS PATHOG	57.78	2.25	2.17	1.96	656
41	PLOS ONE	57.63	2.15	2.09	1.92	2,057
42	J BIOCHEM	57.56	2.05	2.01	1.88	279
43	PROTEIN SCI	57.42	2.07	2.02	1.85	2,235
44	BMC STRUCT BIOL	57.34	2.08	2.02	1.84	228
45	PROTEINS	57.07	2.07	2.01	1.84	1,999
46	J MED CHEM	56.70	2.07	2.02	1.86	5,525
47	BIOPHYS J	56.68	1.92	1.85	1.66	199
48	NAT COMMUN	56.45	2.21	2.11	1.86	3,538
49	BIOCHIM BIOPHYS ACTA	56.31	2.09	2.04	1.87	600
50	ACS MED CHEM LETT	56.01	2.12	2.06	1.88	1,062
51	J BIOL CHEM	55.60	2.12	2.06	1.89	11,055
52	MOL MICROBIOL	55.38	2.16	2.09	1.87	412
53	INT J BIOL MACROMOL	55.06	2.05	2.00	1.80	168
54	CELL HOST MICROBE	54.93	2.50	2.43	2.20	107
55	BIOORG MED CHEM	54.79	2.10	2.04	1.88	659
56	J MOL BIOL	54.52	2.13	2.06	1.88	9,507
57	NAT MICROBIOL	54.48	2.32	2.25	2.04	111
58	STRUCTURE	54.37	2.20	2.11	1.86	5,348
59	J STRUCT BIOL	54.16	2.15	2.08	1.90	1,038
60	J EXP MED	53.69	2.34	2.28	2.08	121

61	BIOCHEM BIOPHYS RES COMMUN	53.40	2.18	2.11	1.92	976
62	PLANT CELL	53.01	2.21	2.17	2.05	138
63	J STRUCT FUNCT GENOM	52.69	2.07	2.03	1.92	168
64	J BACTERIOL	52.09	2.22	2.16	2.01	371
65	J VIROL*	51.02	2.34	2.26	2.06	957
66	MABS	50.71	2.35	2.29	2.14	115
67	NUCLEIC ACIDS RES*	50.60	2.28	2.21	1.98	2,127
68	PNAS*	50.16	2.27	2.19	1.94	7,376
69	MOL PHARMACOL	50.14	2.35	2.27	2.02	129
70	J IMMUNOL	50.07	2.26	2.19	2.02	296
71	PROTEIN CELL	49.74	2.26	2.21	2.04	178
72	PLOS BIOL*	48.78	2.35	2.25	2.01	336
73	RNA	48.30	2.44	2.32	1.97	245
74	BIOORG MED CHEM LETT*	48.16	2.19	2.16	2.05	1,590
75	VIROLOGY	47.40	2.44	2.37	2.18	126
76	CELL REP*	47.07	2.52	2.42	2.16	399
77	EMBO REP	47.02	2.35	2.28	2.09	211
78	SCI ADV	46.77	2.33	2.22	1.85	182
79	ELIFE*	46.16	2.41	2.30	2.03	869
80	CELL RES*	45.08	2.42	2.36	2.20	189
81	GENES DEV*	44.76	2.41	2.33	2.11	279
82	EMBO J*	44.45	2.37	2.30	2.10	1,910
83	COMMUN BIOL*	44.39	2.39	2.31	2.00	104
84	NAT STRUCT MOL BIOL*	44.31	2.40	2.31	2.07	2,915
85	NEURON*	43.47	2.63	2.41	2.08	149
86	NATURE*	43.42	2.52	2.42	2.12	3,060
87	NAT IMMUNOL*	43.10	2.52	2.45	2.22	119
88	IMMUNITY*	42.95	2.44	2.37	2.18	265
89	SCIENCE*	42.78	2.50	2.39	2.10	1,949
90	MOL CELL*	42.05	2.45	2.37	2.14	1,599
91	CELL*	39.37	2.54	2.45	2.20	1,563

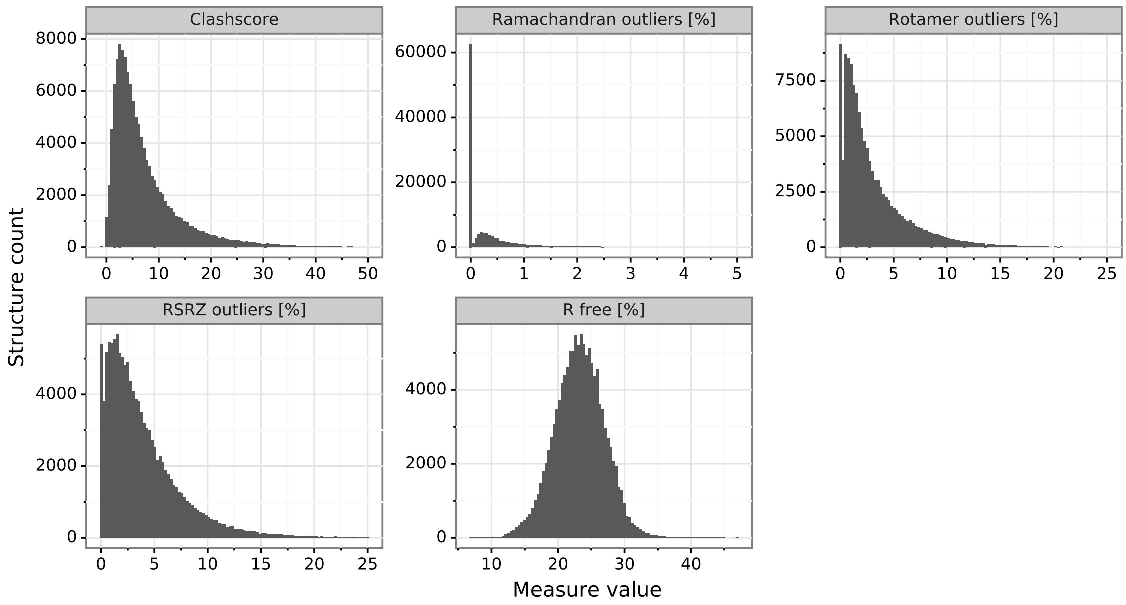

Figure S1. Histograms of quality metric values of structures found in the PDB. The histograms show the distribution of Clashscore, percent of Ramachandran outliers, percentage of rotamer outliers, percentage of RSRZ outliers and R_{free} values. The y-axis shows the number of depositions with a given metric value displayed on the x-axis.

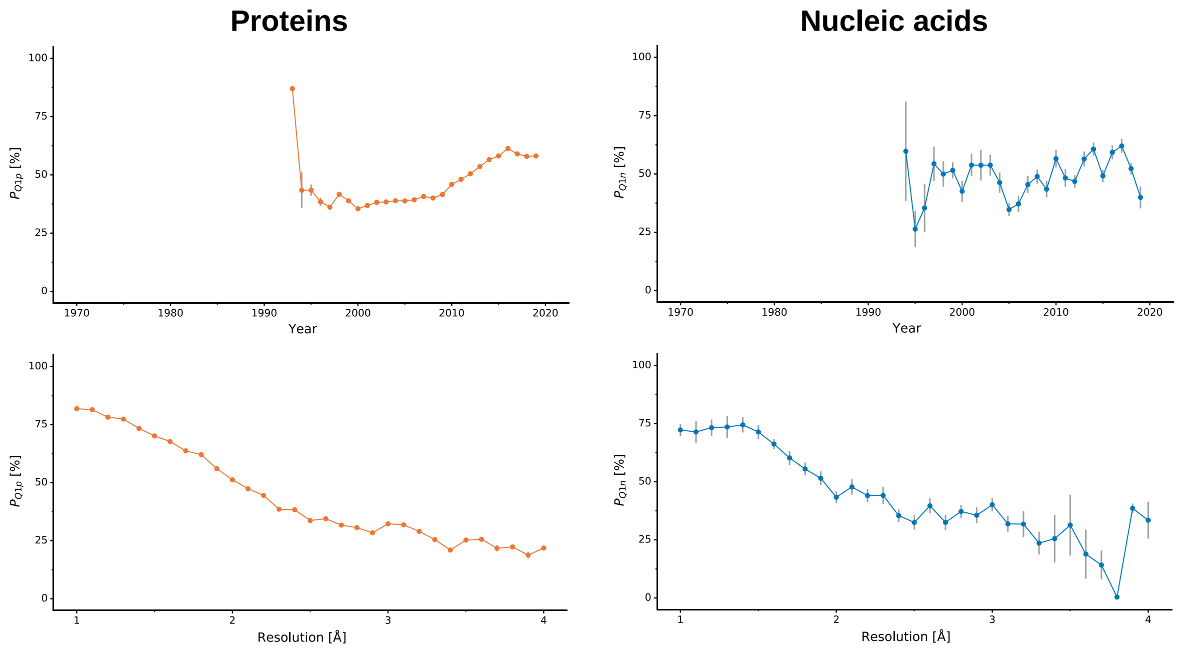

Figure S2. P_{Q1} analysis without imputed values. Variation in the mean P_{Q1} percentile (higher is better) for deposits with all quality metrics over time (top) and resolution (bottom) for proteins (left) and nucleic acids (right). Error bars indicate the estimated unbiased standard error of the mean.

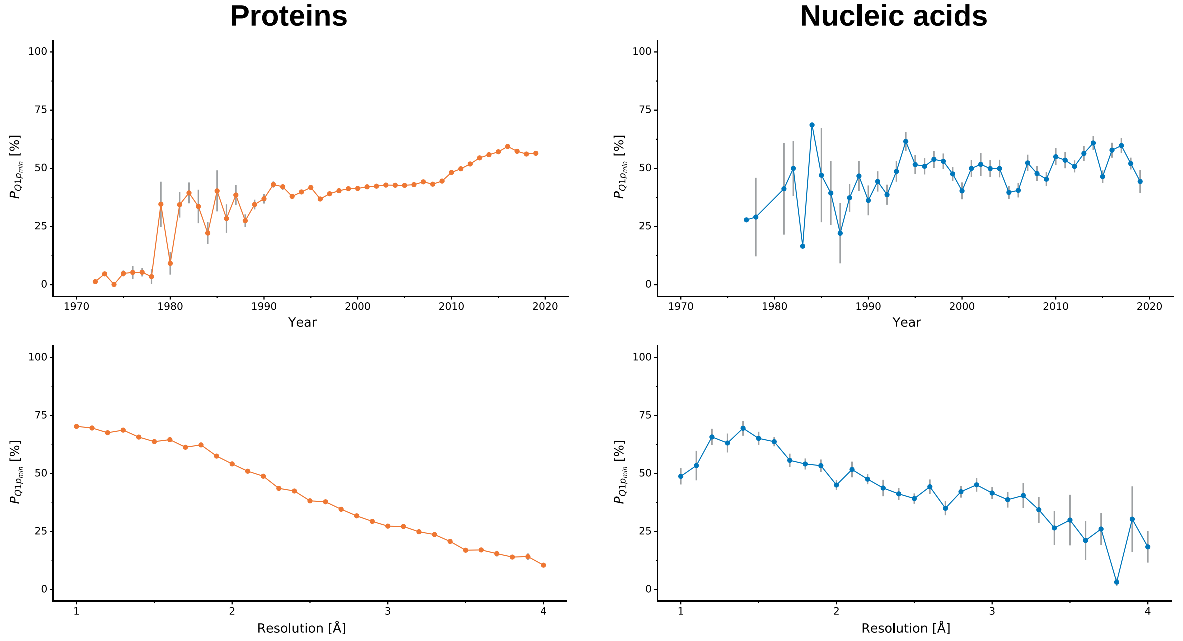

Figure S3. P_{Q1min} analysis (minimum approach). Variation in the mean P_{Q1min} percentile (higher is better) over time (top) and resolution (bottom) for proteins (left) and nucleic acids (right). Error bars indicate the estimated unbiased standard error of the mean.

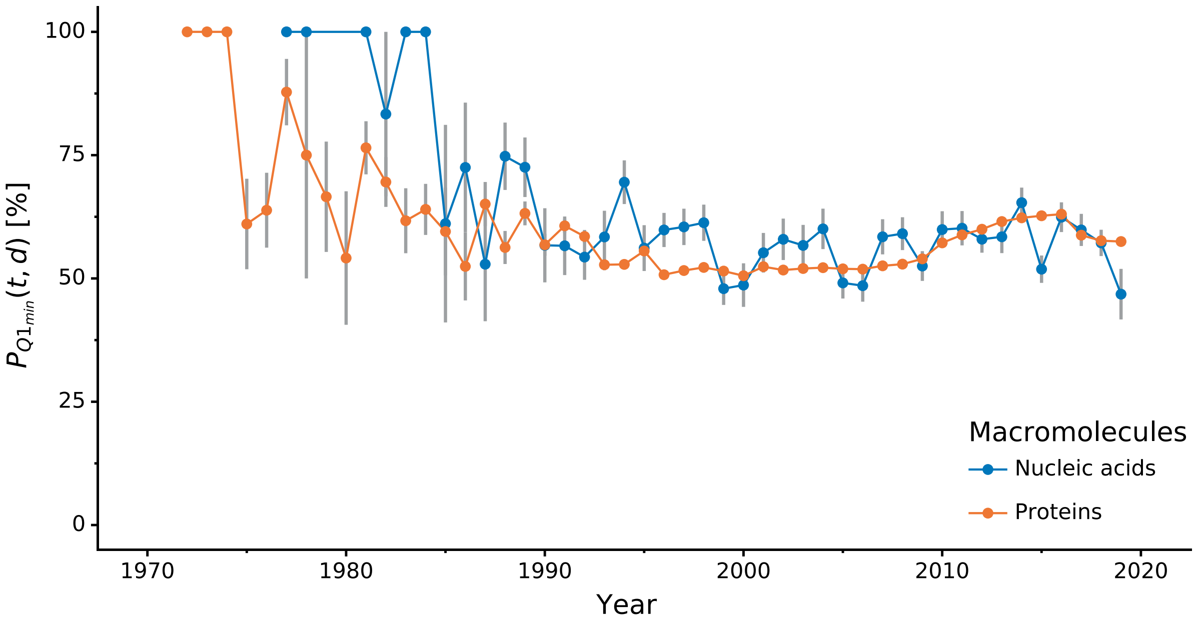

Figure S4. Comparison of $P_{Q1_{min}}(t,d)$ of protein and nucleic acid structures over time. Variation in mean $P_{Q1_{min}}(t,d)$ quality percentile (y-axis, higher is better), comparing nucleic acid and protein structures (color) over time (x-axis). Error bars indicate estimated unbiased standard errors of the mean.

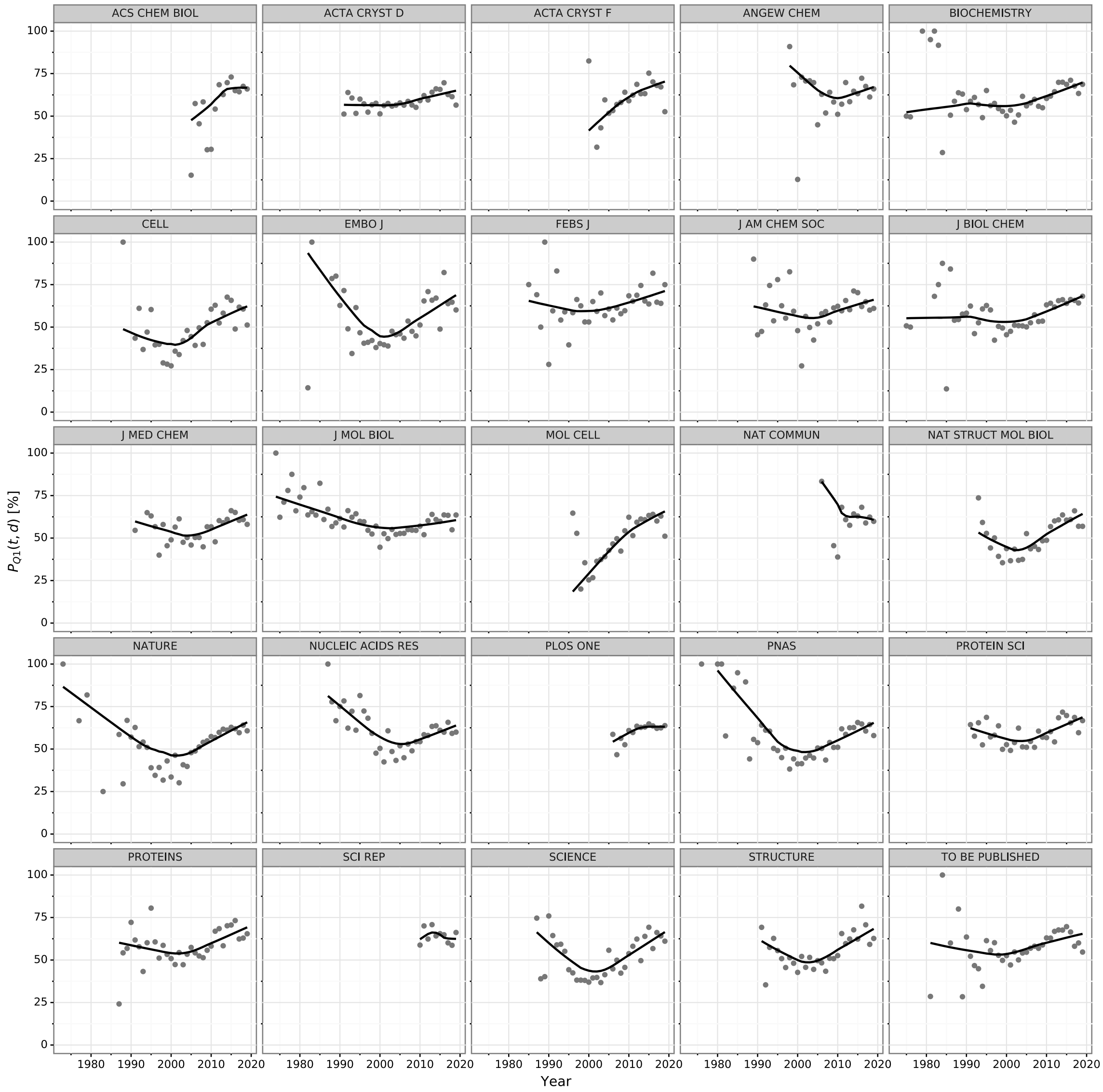

Figure S5. Average $P_{Q1}(t,d)$ of popular journals for each year. The plot shows yearly (x-axis) means of $P_{Q1}(t,d)$ values (y-axis) for each of the 25 most popular journals (panels). Regression lines computed using the locally estimated scatterplot smoothing (LOESS) method.

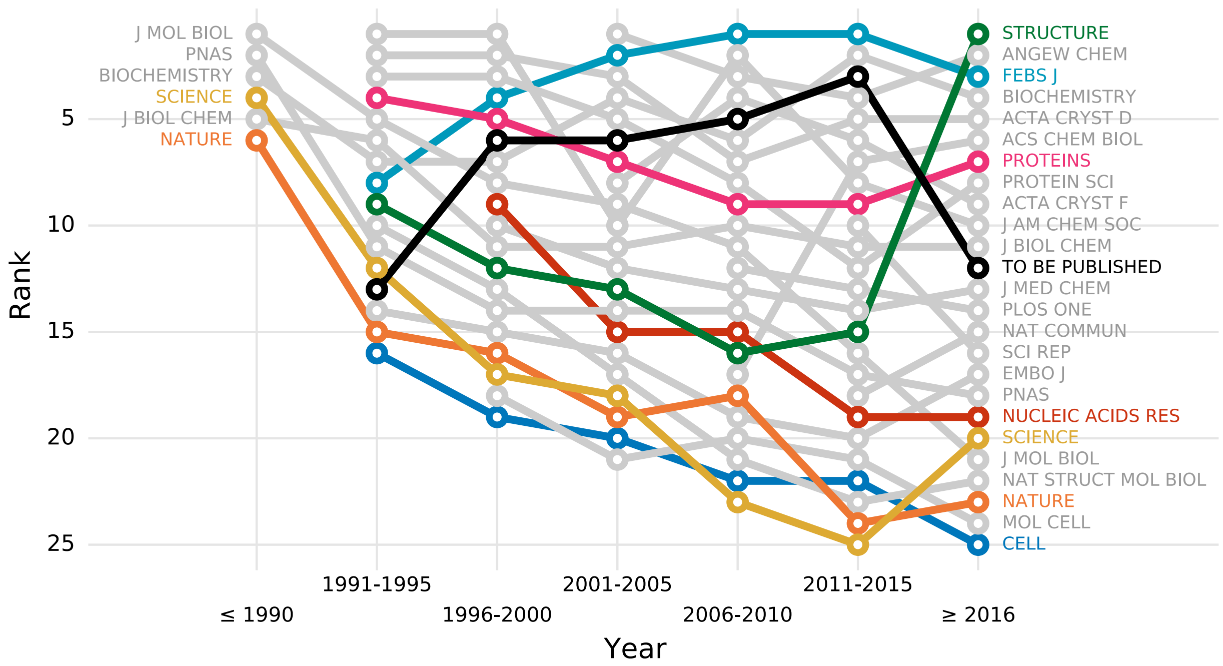

Figure S6. Journal ranking over time according to *$P_{Q1}(t)$*. The plot shows the journal's rank (y-axis) in a given time period (x-axis). Only the 25 most popular journals with at least 30 deposits in a given period were plotted.

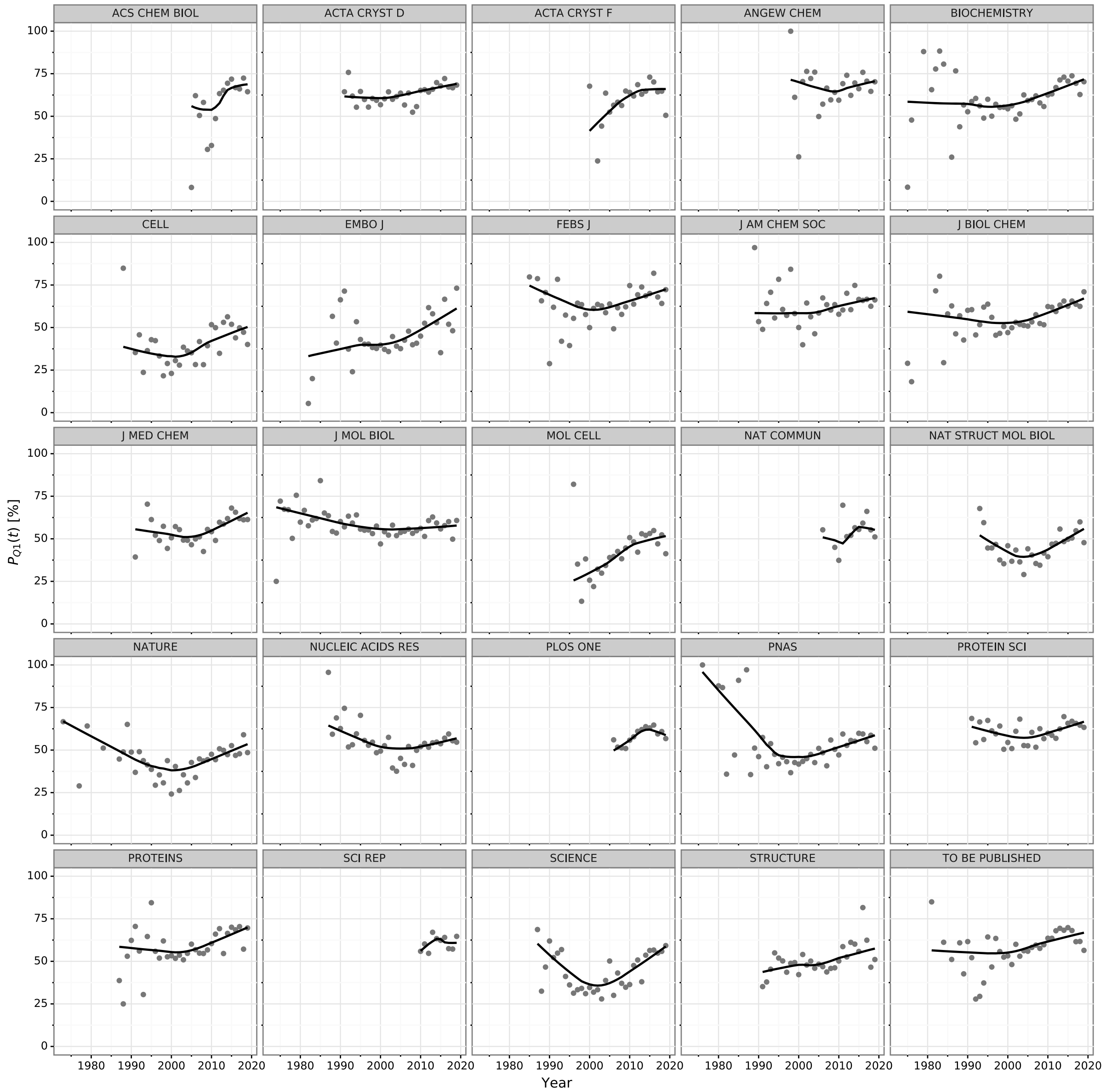

Figure S7. Journal quality over time according to $P_{Q1}(t)$. The plot shows yearly (x-axis) means of $P_{Q1}(t)$ values (y-axis) for each of the 25 most popular journals (panels). Regression lines computed using the locally estimated scatterplot smoothing (LOESS) method.

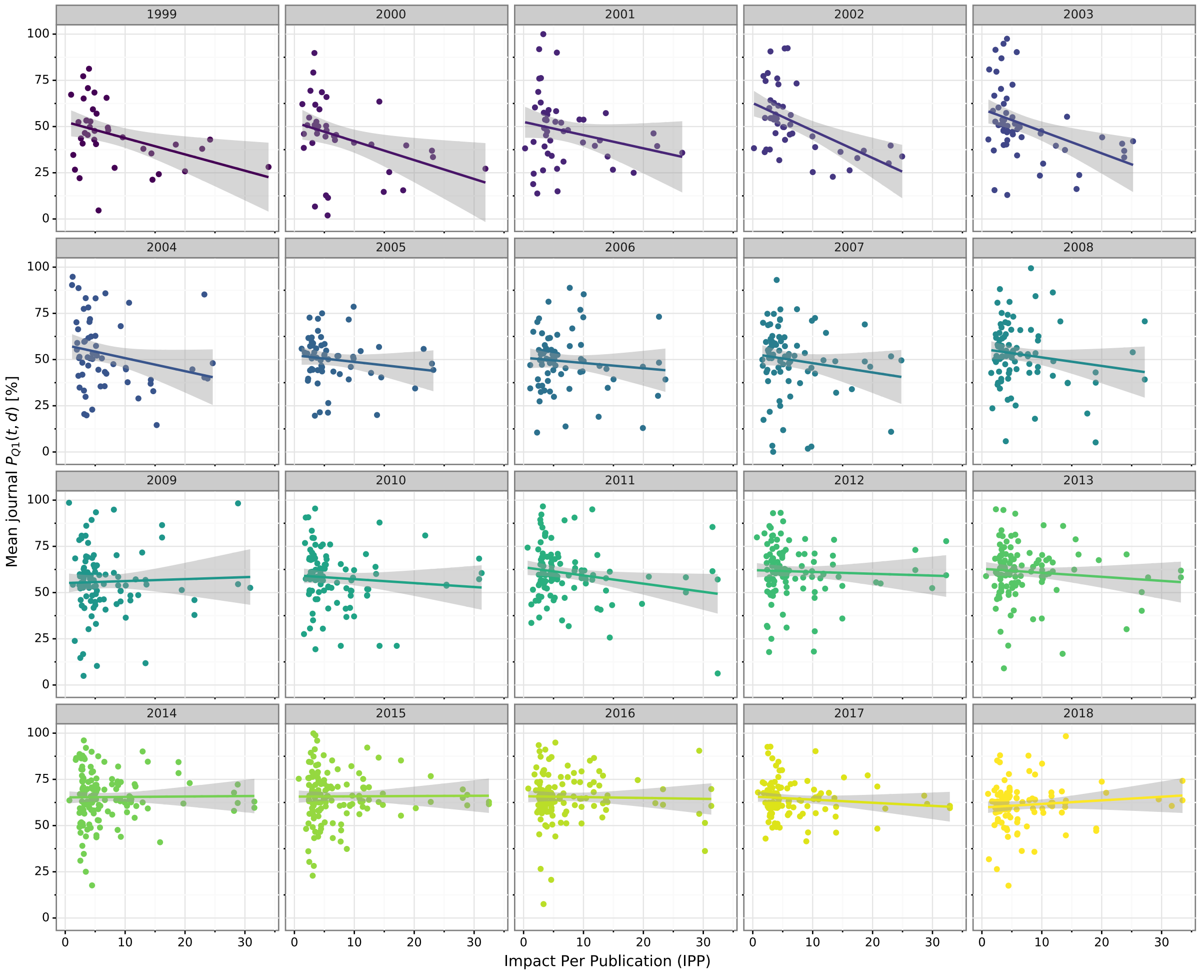

Figure S8. Scatterplots of mean journal $P_{Q1}(t,d)$ and the journal's impact. Variation in mean journal $P_{Q1}(t,d)$ (y-axis) in a given year (panel, color) plotted against the journals Impact Per Publication (IPP). IPP uses the same formula as the 3-year Impact Factor, but is based on publicly available Scopus data. The two regression lines show linear trends with 95% confidence intervals (gray areas).

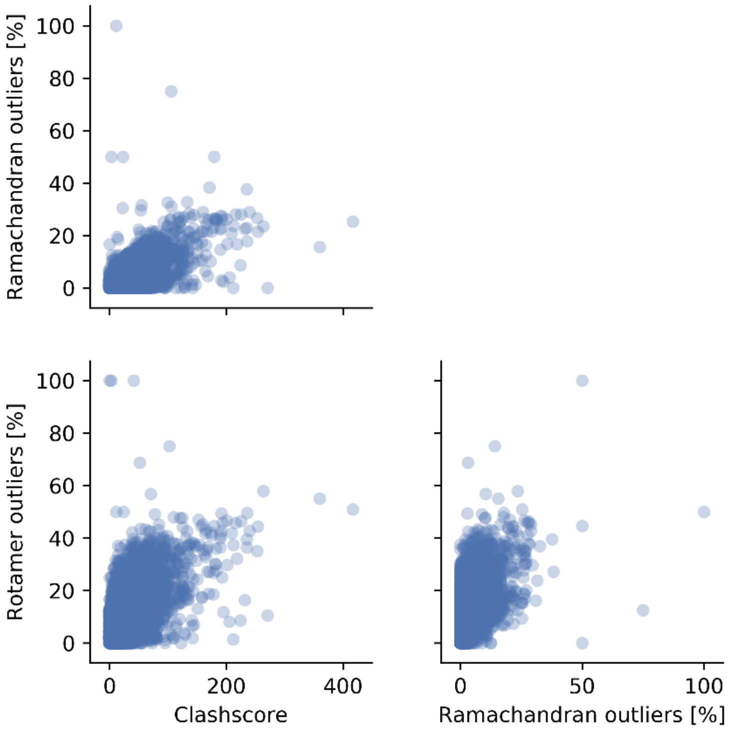

Figure S9. Scatterplots of the values of Clashscore, Ramachandran outliers, and Rotamer outliers found in the PDB. Each point represents a PDB deposit.
